# s-SMOOTH: Sparsity and Smoothness Enhanced EEG Brain Tomography

**DOI:** 10.3389/fnins.2016.00543

**Published:** 2016-11-28

**Authors:** Ying Li, Jing Qin, Yue-Loong Hsin, Stanley Osher, Wentai Liu

**Affiliations:** ^1^Biomimetic Research Lab, Department of Bioengineering, University of California, Los AngelesLos Angeles, CA, USA; ^2^Department of Mathematical Sciences, Montana State UniversityBozeman, MT, USA; ^3^Department of Neurology, Chung Shan Medical UniversityTaichung, Taiwan; ^4^Department of Mathematics, University of California, Los AngelesLos Angeles, CA, USA; ^5^California NanoSystems Institute, University of California, Los AngelesLos Angeles, CA, USA

**Keywords:** EEG source imaging, inverse problem, total generalized variation (TGV), ℓ_1−2_ regularization, difference of convex functions algorithm (DCA), alternating direction method of multipliers (ADMM)

## Abstract

EEG source imaging enables us to reconstruct current density in the brain from the electrical measurements with excellent temporal resolution (~ *ms*). The corresponding EEG inverse problem is an ill-posed one that has infinitely many solutions. This is due to the fact that the number of EEG sensors is usually much smaller than that of the potential dipole locations, as well as noise contamination in the recorded signals. To obtain a unique solution, regularizations can be incorporated to impose additional constraints on the solution. An appropriate choice of regularization is critically important for the reconstruction accuracy of a brain image. In this paper, we propose a novel Sparsity and SMOOthness enhanced brain TomograpHy (s-SMOOTH) method to improve the reconstruction accuracy by integrating two recently proposed regularization techniques: Total Generalized Variation (TGV) regularization and ℓ_1−2_ regularization. TGV is able to preserve the source edge and recover the spatial distribution of the source intensity with high accuracy. Compared to the relevant total variation (TV) regularization, TGV enhances the smoothness of the image and reduces staircasing artifacts. The traditional TGV defined on a 2D image has been widely used in the image processing field. In order to handle 3D EEG source images, we propose a voxel-based Total Generalized Variation (vTGV) regularization that extends the definition of second-order TGV from 2D planar images to 3D irregular surfaces such as cortex surface. In addition, the ℓ_1−2_ regularization is utilized to promote sparsity on the current density itself. We demonstrate that ℓ_1−2_ regularization is able to enhance sparsity and accelerate computations than ℓ_1_ regularization. The proposed model is solved by an efficient and robust algorithm based on the difference of convex functions algorithm (DCA) and the alternating direction method of multipliers (ADMM). Numerical experiments using synthetic data demonstrate the advantages of the proposed method over other state-of-the-art methods in terms of total reconstruction accuracy, localization accuracy and focalization degree. The application to the source localization of event-related potential data further demonstrates the performance of the proposed method in real-world scenarios.

## 1. Introduction

Functional brain imaging techniques have been developed to evaluate brain function, e.g., memory and cognition, as well as help diagnose and treat brain disorders, e.g., epilepsy, depression, schizophrenia and Alzheimer's disease. Ideally, a good imaging technique needs to provide brain image of both high temporal and high spatial resolution. Hemodynamic imaging techniques such as functional Magnetic Resonance Imaging (fMRI) and Positron Emission Tomography (PET) have been widely used since they offer high spatial resolution (Poldrack and Sandak, 2004). However, their temporal resolution is limited on the order of seconds due to the relatively slow blood flow response (Poldrack and Sandak, 2004). Furthermore, these imaging systems require the subject to be restricted in a large chamber, which limits their applications in the natural habitual environment. On the other hand, brain imaging based on electroencephalography (EEG) provides an alternative solution that overcomes these limitations. Unlike fMRI and PET, EEG has much higher temporal resolution in the range of milliseconds. In addition, it is lightweight and portable, hence can be used in various applications that require natural environments, such as learning in a classroom. Nevertheless, EEG source imaging suffers from relatively low reconstruction accuracy due to the ambiguity of the underlying inverse problem (Baillet et al., 2001). To mitigate this disadvantage, appropriate constraints could be incorporated into EEG inverse problem to improve reconstruction accuracy of the brain image.

In general, there are two types of models for EEG source imaging: dipolar and distributed source model (Michel et al., 2004). The dipolar model (Sidman et al., 1978; Scherg and Von Cramon, 1986; Mosher et al., 1992) assumes that a small number of focal sources are active so only a few parameters of these sources need to be estimated. Since the number of unknown parameters is usually smaller than that of the measurements, the corresponding inverse problem is over-determined and can be solved by non-linear optimization techniques (Uutela et al., 1998). However, the source reconstruction is usually highly sensitive to the initial values due to the high non-convexity of the objective function. Furthermore, this model is not able to handle the spatially extended sources, such as that during the propagation of a seizure. On the other hand, in the distributed source model (Hämäläinen and Ilmoniemi, 1984; Hämäläinen et al., 1993), the source space is divided into a lot of voxels with fixed locations, and only the activation in each location needs to be estimated. However, due to a relatively small number of electrodes (~10^2^) and a large number of potential dipole locations (~10^4^), the corresponding inverse problem is highly under-determined and results in infinitely many solutions. To obtain a unique solution, regularization can be used to impose additional constraints on the solution. The conventional minimum ℓ_2_-norm methods, such as minimum norm estimate (MNE) (Hämäläinen et al., 1993) and standardized low resolution brain electromagnetic tomography (sLORETA) (Pascual-Marqui, 2002), use ℓ_2_-norm of the current density as the regularization term, leading to a solution with minimal energy. These methods usually have a closed-form solution thus the computational cost is relatively low. However, they share a limitation that the reconstructed sources spread over a large area of the brain, resulting in a brain image with low spatial resolution, i.e., proximal sources may become indistinguishable in the solution.

**Figure 1:**
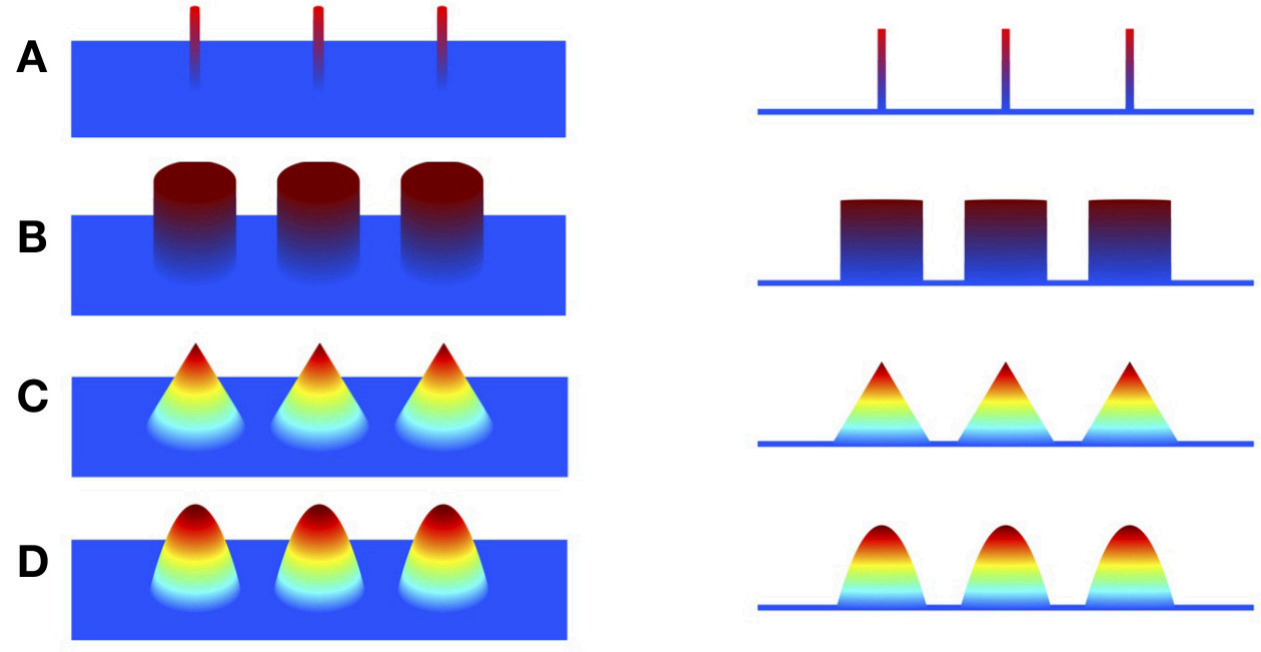
**Illustration of piecewise polynomial current densities in 3D view and side view. (A–D)** Impulse (sparse in itself), piecewise constant (sparse in first spatial derivative), piecewise linear (sparse in second derivative), piecewise quadratic (sparse in third derivative).

To overcome the limitation of minimum ℓ_2_-norm methods, sparse structure of the underlying source is explored to improve the focalization of the source. Minimizing ℓ_1_-norm methods, such as minimum current estimate (MCE) (Uutela et al., 1999) and sparse source imaging (SSI) (Ding and He, 2008), were proposed by employing ℓ_1_-norm of the current density as the regularization, assuming that the source current density is sparse with only a few active voxels (Figure 1A). Although the focalization is greatly improved, these methods fail to estimate the extent of the sources since the reconstructed source is over-focused. To address this issue, efforts have been devoted to exploring sparsity on transform domains of the current density, such as the spatial Laplacian domain (Haufe et al., 2008; Vega-Hernández et al., 2008; Chang et al., 2010), wavelet-basis domain (Chang et al., 2010; Liao et al., 2012; Zhu et al., 2014), Gaussian-basis domain (Haufe et al., 2011), or variation domain (Adde et al., 2005; Ding, 2009; Gramfort, 2009; Luessi et al., 2011; Becker et al., 2014; Sohrabpour et al., 2016). Furthermore, in order to obtain a local smooth and global sparse result, some approaches impose sparsity on both the transform domain and the original source domain. For example, Focal Vector field Reconstruction (FVR) (Haufe et al., 2008) and ComprEssive Neuromagnetic Tomography (CENT^*L*^) (Chang et al., 2010) impose sparsity on the spatial Laplacian and the current density itself. It has been shown that combination of these two regularization terms improves the imaging results than using ℓ_2_-norm or ℓ_1_-norm regularization alone. However, the Laplacian operator, i.e., the sum of all unmixed second partial derivatives, tends to assign high weight to the central voxel and relatively low weights to its neighbors, which results in the over-smoothing effect of the reconstructed image (refer to Section 4). Sparse Total Variation (TV) methods, also known as TV-ℓ_1_ (Becker et al., 2014; Sohrabpour et al., 2016), impose the sparsity constraint on both the spatial gradient and the current density itself. They assume that the current density distribution is piecewise constant, and are able to preserve well the extent of the sources. However, due to the piecewise constant assumption, the reconstructed current density distribution is almost uniform in each subregion (so called “staircasing effect”), which fails to reflect the intensity variation of the source in space. As a consequence, these methods have difficulty localizing peaks of the source, leading to relatively large localization error.

The present study aims at reconstructing the location, extent and magnitude variation of spatially extended sources with high accuracy by employing more advanced regularization techniques. We adopt the strategy that promotes global sparsity and local smoothness simultaneously, and propose a Sparsity and SMOOthness enhanced brain TomograpHy (s-SMOOTH) method to improve reconstruction accuracy. More specifically, a voxel-based Total Generalized Variation (vTGV) regularization is employed to promote sparsity on the spatial derivative, and the ℓ_1−2_ regularization is utilized to impose sparsity on the current density itself. The total generalized variation (TGV) regularization (Bredies et al., 2010) has been shown to outperform the Laplacian-based, wavelet-based and TV-based regularizations in compressive sensing MRI reconstruction (Knoll et al., 2011; Qin and Guo, 2013; Guo et al., 2014), image deconvolution and denoising (Bredies et al., 2010; Qin et al., 2014). Comparing to the TV, the TGV incorporates information of higher-order derivatives, and therefore is better suited for modeling piecewise smooth functions (Benning et al., 2013; Bredies and Holler, 2014). Notice that the traditional TGV is defined on a 2D image and its extension to an irregular surface is challenging. In order to deal with the 3D cortex surface, we define a voxel-based TGV (vTGV) regularization which extends the definition of the second order TGV from 2D image to an irregular triangular mesh such as the cortical surface. vTGV enhances the smoothness of the brain image and reconstructs the spatial distribution of the current density more precisely. Meanwhile, motivated by the performance of the ℓ_1−2_ regularization in compressive sensing reconstruction and other image processing problems (Esser et al., 2013; Lou et al., 2014; Yin et al., 2015), we incorporate the ℓ_1−2_ regularization into the objective function. Numerical experiments show that ℓ_1−2_ regularization provides faster convergence and yields sparser source image than the ℓ_1_-norm regularization. Furthermore, by applying the difference of convex function algorithm (DCA) and alternating direction method of multipliers (ADMM), we derive an efficient numerical algorithm to solve the corresponding optimization problem. A variety of simulation tests on Gaussian-shaped sources with various noise levels, source sizes, source configurations and locations show that the proposed approach results in better performance than the state-of-the-art methods in terms of total reconstruction accuracy, localization accuracy and focalization degree. The tests on auditory and visual P300 data further demonstrate that the proposed method is able to preserve high order smoothness and produce brain images with higher spatial resolution.

The paper is organized as follows. Section 2 introduces the EEG inverse problem and describes the proposed s-SMOOTH method based on the vTGV and ℓ_1−2_ regularizations. In Section 3, we show a series of experimental results using synthetic data and real data, and compare various methods qualitatively and quantitatively. Finally, the results and future directions are discussed in Section 4 and a brief conclusion is drawn in Section 5.

## 2. Materials and methods

### 2.1. EEG inverse problem

As a non-invasive method, electroencephalography (EEG) is used to measure brain activity and detect abnormalities associated with certain brain disease. When neurons in the brain are activated, local currents are generated, and can travel through different tissues, e.g., gray matter, cerebrospinal fluid (CSF), skull and scalp. These currents result in electrical potentials on the scalp that are recorded by electrodes as the EEG signals. The EEG inverse problem refers to the process of reconstructing the spatial distributions of currents in the form of a 3D brain image given the electrical recordings. To formulate the inverse problem in mathematical expressions, we consider a distributed source model assuming that dipole sources are located on the cortex surface (Dale and Sereno, 1993), which is discretized as a mesh consisting of a large number of small triangles. From now on we treat each triangle as one voxel in the discretized source space, and the terms triangle and voxel are used interchangeably. In addition, we assume that the orientation of each dipole is perpendicular to the cortex surface (Dale and Sereno, 1993). This is based on the assumption that most of the current flow to the scalp is produced by cortical pyramidal cells, which are normal to the cortical surface (Dale and Sereno, 1993; Nunez and Srinivasan, 2006). Let *b* ∈ ℝ^*N*^ be the electrical potential on the scalp measured by the electrodes, where *N* is the number of electrodes, and *u* ∈ ℝ^*M*^ is the neural current density at each dipole location. The electrode potential *b* can be related to the neural current *u* by the following linear equation

(1) b=Au+n,

where *n* ∈ ℝ^*N*^ denotes the noise, and *A* ∈ ℝ^*N*×*M*^ is called *lead field matrix*. Note that the (*i, j*)-th entry of *A* stands for the electrical potential measured by the *i*th electrode due to a unit dipole source at the *j*th voxel. The matrix *A* can be calculated by constructing a head model (Oostendorp and van Oosterom, 1991; Gulrajani, 1998; Fuchs et al., 2002), and solving the Maxwell′s equations (Sarvas, 1987) with the boundary element method (BEM) (Oostendorp and van Oosterom, 1991; Fuchs et al., 2002). Usually the number of voxels *M* is much larger than the number of electrodes *N*, thus the linear system Equation (1) is highly under-determined and has infinitely many solutions. To guarantee uniqueness of the solution for the distributed source model, regularization techniques can be applied to impose additional constraints on the solution. We consider the following model to reconstruct the brain image

(2) $$\underset{u}{\min}\frac{1}{2}∥Au-b{∥}_{2}^{2}+\;\alpha R(u),$$

Here the first term, called data fidelity term, reflects the statistics of the Gaussian noise. The second term is the regularization term which is related to the assumption on the characteristics of *u*, e.g., smoothness or sparsity.

### 2.2. s-SMOOTH

In this section, we will first briefly review the definition of the second order TGV on a 2D-grid image, and then define vTGV on a triangular mesh such as the discretized cortex surface. After that, the ℓ_1−2_ regularization will be introduced. Finally, Section 2.2.4 will describe the proposed model and derive an efficient algorithm to solve the optimization problem. The parameter selection for the algorithm will also be discussed.

#### 2.2.1. Total generalized variation

TGV was proposed to preserve high order of smoothness in image processing problems (Bredies et al., 2010). Based on the assumption that the underlying image is piecewise polynomial, TGV exploits sparsity of high order derivatives along the *x*-axis and the *y*-axis. For the illustrative purpose, we display in Figure 1 various piecewise polynomials defined on a plane with degree up to two. Given a 2D image *u* twice continuously differentiable on a bounded set $\bar{\Omega }\subset {\mathbb{R}}^{2}$, the second order TGV of *u* with the coefficient α = (α_1_, α_2_) can be defined as the following infimal convolution (Bredies et al., 2010; Guo et al., 2014)

(3) $${\text{TGV}}_{\alpha }^{2}(u)=\underset{p=(p_{1},p_{2})\in {\left(C^{2}(\bar{\Omega },\mathbb{R})\right)}^{2}}{\min}\alpha_{1}\|\nabla u-p{\|}_{1}+\alpha_{2}\|\tilde{ℰ}(p){\|}_{1},$$

where ∇ is the 2D gradient operator, *p* is an auxiliary variable, and the operator $\tilde{ℰ}$ is defined by

(4) $$\tilde{ℰ}(p)=\left[\begin{array}{cc} \frac{\partial p_{1}}{\partial x} & \frac{1}{2}(\frac{\partial p_{2}}{\partial x}+\frac{\partial p_{1}}{\partial y})\\ \frac{1}{2}(\frac{\partial p_{2}}{\partial x}+\frac{\partial p_{1}}{\partial y}) & \frac{\partial p_{2}}{\partial y} \end{array}\right].$$

Here the ℓ_1_-norm of a matrix treats a matrix as a vector, i.e., $\left\|{X\|}_{1}=\underset{i,j}{\sum }|X_{i,j}\right|$. Different from the Laplacian operator which only involves all unmixed second partial derivatives, the second-order TGV involves all partial derivatives, similar to Hessian. In (3), when ∇*u* is equal to *p*, the first term in the objective function becomes zero and $\tilde{ℰ}$ becomes the Hessian of *u*. Therefore, one can see that $TGV\left(u\right)\leq {\|H\left(u\right)}_{1}$ where H(u) is the Hessian of *u*. This suggests that TGV could yield a faster minimizing sequence than ${\left\|H\left(u\right)\right\|}_{1}$, therefore it is a better choice as a regularization term for imposing sparsity than the ℓ_1_-norm of Hessian in terms of convergence rate.

#### 2.2.2. Voxel-based total generalized variation for smoothness enhancement

**Figure 2:**
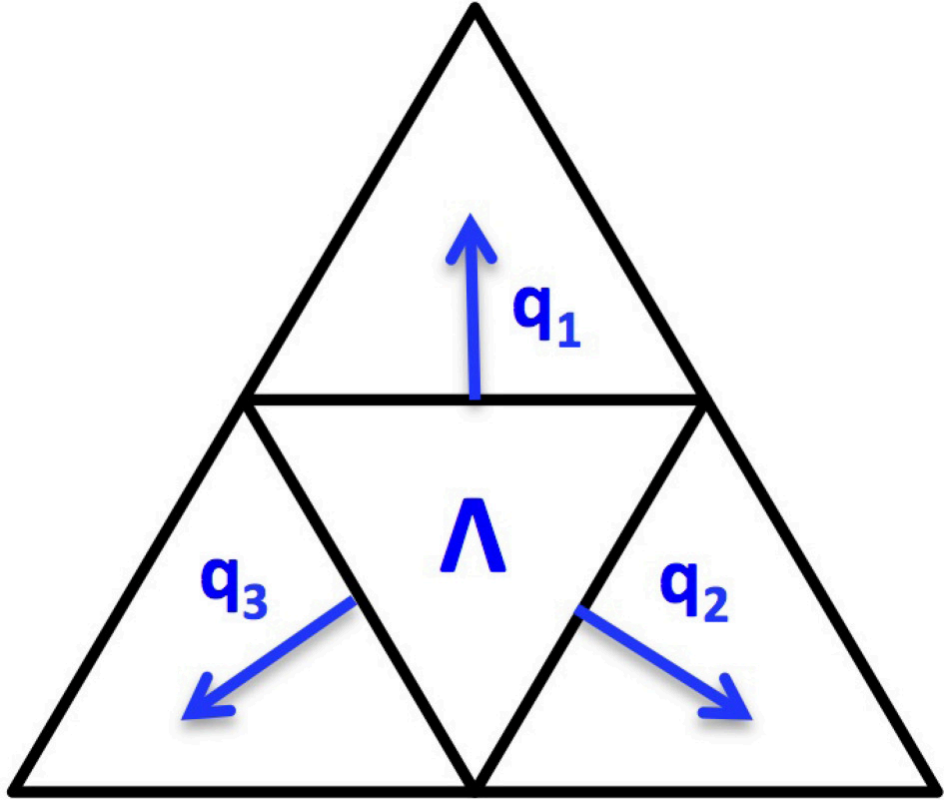
**Illustration of three normal directions to a triangular voxel Λ**.

Since the cortex surface has complicated geometries and topological structures, it is crucial to choose an appropriate regularization tailored to such kind of irregular surfaces. We discretize the cortex surface to be a 3D triangular mesh Ω and define a voxel-based TGV (vTGV) regularization on it. In order to define directional derivatives on triangular mesh, we treat the centroid of each triangular voxel as a dipole. Since each voxel has three voxels connected, three directional derivatives on ℝ^3^ can be used to define “gradient” of the density function *u*. Consider a triangular voxel Λ ∈ Ω, which is homeomorphic to ℝ^2^, we assume that *q*_1_, *q*_2_, *q*_3_ are three normal directions along three edges for Λ, where $q_{i}\in {\mathbb{R}}^{3}$ depends on the shape of the triangle Λ. For instance, Figure 2 illustrates three normal directions associated with a triangular voxel. Although not perpendicular to each other, these three directions can span the tangent plane through each voxel and thereby can be used to fully describe variations of *u*. The gradient of *u* restricted on Λ is defined by

(5) $$\hat{\nabla }u=\left[\begin{array}{c} \frac{\partial u}{\partial q_{1}}\\ \frac{\partial u}{\partial q_{2}}\\ \frac{\partial u}{\partial q_{3}} \end{array}\right],\;\frac{\partial u}{\partial q_{i}}=\underset{\begin{array}{c} h\to 0\\ x,x+hq_{i}\in \Lambda \end{array}}{\lim}\frac{u(x+hq_{i})-u(x)}{h}.$$

Note that this definition is in the local sense and it can be considered as an extension of the gradient operator in ℝ^2^ into the gradient in a 2D manifold. Given a differentiable function *p* = (*p*_1_, *p*_2_, *p*_3_), the operator ℰ acting on *p* restricted to Λ is defined by

(6) $$ℰ(p)=\left[\begin{array}{ccc} \frac{\partial p_{1}}{\partial q_{1}} & \frac{1}{2}(\frac{\partial p_{2}}{\partial q_{1}}+\frac{\partial p_{1}}{\partial q_{2}}) & \frac{1}{2}(\frac{\partial p_{3}}{\partial q_{1}}+\frac{\partial p_{1}}{\partial q_{3}})\\ \frac{1}{2}(\frac{\partial p_{1}}{\partial q_{2}}+\frac{\partial p_{2}}{\partial q_{1}}) & \frac{\partial p_{2}}{\partial q_{2}} & \frac{1}{2}(\frac{\partial p_{3}}{\partial q_{2}}+\frac{\partial p_{2}}{\partial q_{3}})\\ \frac{1}{2}(\frac{\partial p_{1}}{\partial q_{3}}+\frac{\partial p_{3}}{\partial q_{1}}) & \frac{1}{2}(\frac{\partial p_{2}}{\partial q_{3}}+\frac{\partial p_{3}}{\partial q_{2}}) & \frac{\partial p_{3}}{\partial q_{3}} \end{array}\right].$$

This operator can be considered as an extension of $\tilde{ℰ}$ in Equation (4) tailored to the triangular mesh Ω.

Next, we discuss the discretization of the operators $\hat{\nabla }$ and ℰ. On the triangular mesh Ω with *M* voxels, we first index all voxels and then define a finite difference operator matrix *D* ∈ ℝ^3*M*×*M*^ as follows. The (*i, j*)-th entry of *D* is defined as

(7) $$D_{i,j}=\{\begin{array}{ll} 1, & \text{if }j=l;\\ -1, & \text{if }j\in \{k_{l,1},k_{l,2},k_{l,3}\};\\ 0, & \text{otherwise}, \end{array}$$

where the voxel index is *l* = ⌈*i*/3⌉ ∈ {1, …, *M*}, i.e., the smallest integer no less than *i*/3, and *k*_*l*,1_, *k*_*l*,2_ and *k*_*l*,3_ are the indices of the voxels adjacent to the *l*-th voxel. Based on the definition in Equation (6), the discretization of the operator ℰ is defined as *E* ∈ ℝ^3*M*×3*M*^ of the form

(8) $$\begin{array}{l} E=\frac{1}{2}(\hat{D}\;+\;{\hat{D}}^{T}),\text{ where }\hat{D}=I_{1\times 3}\;⊗\;D,\text{ and}\\ \;I_{1\times 3}=\left[\begin{array}{ccc} 1 & 1 & 1 \end{array}\right], \end{array}$$

where ⊗ is the Kronecker product of two matrices. Note that each edge is counted twice in Equation (7) so that the operator *E* can be easily constructed by using *D*. Moreover, *E* is symmetrized by taking the average between $\hat{D}$ and its transpose.

One can see that $\hat{\nabla }u$ is discretized by *Du*, and ℰ(p) is discretized by *Ep*. Once *D* and *E* are available, TV and the second order vTGV with the coefficients α_1_ and α_2_ can be defined as

(9) $$\text{TV}(u)=\|Du{\|}_{1},$$

(10) $${\text{vTGV}}_{\left(\alpha_{1},\alpha_{2}\right)}^{2}(u)=\underset{p\in {\mathbb{R}}^{3M}}{\min}\alpha_{1}\|Du-p{\|}_{1}+\;\alpha_{2}\|Ep{\|}_{1}.$$

In Equation (10), the parameters α_1_ and α_2_ balance the first and second order derivative information of the image (Papafitsoros and Valkonen, 2015). It has been proven that for a large ratio α_2_/α_1_, the second order TGV coincides with TV under certain conditions (Papafitsoros and Valkonen, 2015).

TV is able to well preserve the edges of images, but is known to create piecewise constant result even in regions with smoothly changed intensities (Benning et al., 2013). By considering higher-order derivative information, TGV generalizes TV and is able to reduce staircasing effects by assuming that the image to be reconstructed is piecewise polynomial (including piecewise constant, piecewise linear, piecewise quadratic, etc.)(Bredies and Holler, 2014). In particular, the proposed second order vTGV assumes that the underlying current density distribution is piecewise linear, and thereby this regularization is able to enforce the sparsity of second spatial derivatives. Although a natural image may have higher order smoothness, it is usually sufficient to use the second order vTGV in practice, since performance enhancement is limited but more computations are required for higher order vTGV. Therefore, we only use the second order vTGV regularization in this work.

#### 2.2.3. ℓ_1−2_ regularization for sparsity enhancement

In order to improve the spatial resolution of the brain image to better separate close sources, we can incorporate sparsity constraint into the model. A natural strategy to impose sparsity is ℓ_0_-norm regularization which minimizes the number of non-zero intensity values in the image. However, since the ℓ_0_-regularized problem is computational NP-hard, its ℓ_1_-norm relaxed version is usually considered in practice. Recently ℓ_1−2_ regularization has been proposed (Esser et al., 2013; Lou et al., 2014; Yin et al., 2014), and has been shown to provide a sparser result than the widely used ℓ_1_-norm regularization.

For a real positive number *p*, the ℓ_*p*_-norm of *u* ∈ ℝ^𝕄^ is defined as

(11) $$\|u{\|}_{p}={\left(\sum_{i=1}^{M}|u_{i}{|}^{p}\right)}^{\frac{1}{p}},\;p>0.$$

Different from the ℓ_*p*_-norm, the ℓ_1−2_ regularization penalty function is defined as

(12) $$\|u{\|}_{1-2,\beta }=\|u{\|}_{1}-\beta \|u{\|}_{2},\;0<\beta \leq 1,$$

which has shown potential in image processing and compressive sensing reconstruction (Lou et al., 2014; Yin et al., 2015) in terms of sparsity and fast convergence. It promotes sparsity of an image, and achieves the smallest value when only one voxel in the image is non-zero.

**Figure 3:**
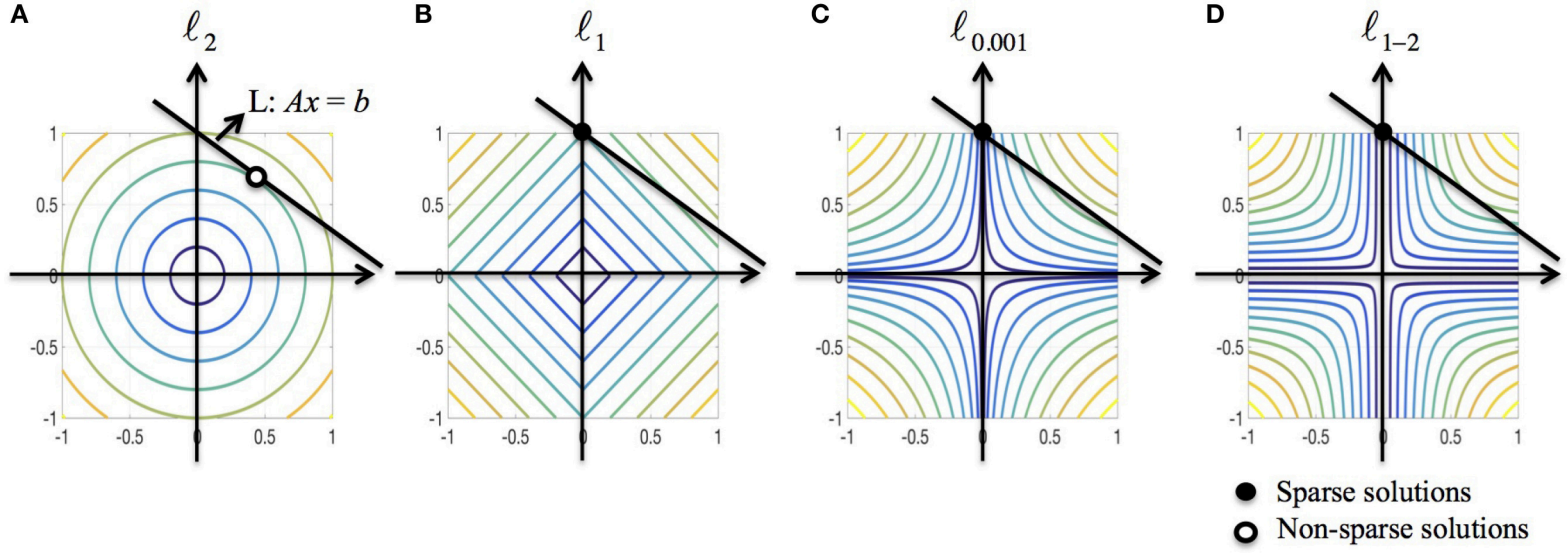
**Geometric interpretation of sparsity for various regularizations. (A–D)** ℓ_2_, ℓ_1_, ℓ_0.001_ (used to approximate ℓ_0_), and ℓ_1–2_ when β = 1. The black line corresponds to the linear constraint, the solid dot specifies the sparse solution and the circular dot specifies the non-sparse solution.

We further discuss the sparsity property of ℓ_1−2_ regularization from the optimization point of view. Consider a minimization problem in 2D $\underset{x\in {\mathbb{R}}^{2}}{\min}R\left(x\right)$ subject to the linear constraint *Ax* = *b* where *R*(*x*) is a regularization function. To solve the problem graphically, we need to find the level curve of minimum radius to the origin that intersects with the line *L* : *Ax* = *b*. Figure 3 illustrates the solutions when *R* is ℓ_2_, ℓ_1_, ℓ_0.001_ (used to approximate ℓ_0_) and ℓ_1−2_ when β = 1, respectively. As shown in Figure 3A, the ℓ_2_-regularized solution rarely has zero components, indicating that the solution is usually non-sparse. The ℓ_1_-regularized solution may not be sparse if the line *L* is parallel to the level curves. Compared to ℓ_*p*_ (0 < *p* < 1) regularization, the ℓ_1−2_ regularization is more likely to yield a sparse solution due to the curvature of level curves. Therefore, the ℓ_1−2_ regularization promotes sparser solutions than the other regularizations being compared. In the EEG inverse problem, the brain images to be reconstructed in general have a sparse structure that the number of sources is limited, which motivates us to apply the ℓ_1−2_ regularization to solve this problem.

In this paper, we unify the ℓ_1_ type and the ℓ_1−2_ type regularizations by allowing β = 0 in Equation (12), so that the sparsity regularization term could be adjusted by tuning the parameter β.

#### 2.2.4. Proposed EEG reconstruction algorithm

The following model is proposed to reconstruct the EEG brain image *u*

(13) $$\underset{u}{\min}\frac{1}{2}\|Au-b{\|}_{2}^{2}+{\text{vTGV}}_{\left(\alpha_{1},\alpha_{2}\right)}^{2}(u)+\alpha_{3}\|u{\|}_{1-2,\beta },$$

where ${vTGV}_{\left(\alpha_{1},\alpha_{2}\right)}^{2}\left(u\right)$ is defined in Equation (10), and ‖*u*‖_1−2,β_ is defined in Equation (12). Here α_*i*_ > 0 are regularization parameters which control the contribution of each regularization term. Note that if β = 0, the ℓ_1−2_ regularization reduces to the ℓ_1_ regularization. If we require *p* = **0**, then the vTGV regularization reduces to the TV.

Since the dual norm of ‖·‖_2_ is itself, i.e., ${\left\|u\right\|}_{2}=\underset{q_{2}\leq 1}{\left\|\max\right\|}\langle u,q\rangle$, the model Equation (13) can be reformulated as

(14) $$\begin{array}{l} \underset{u,p,\|q{\|}_{2}\leq 1}{\min}\frac{1}{2}\|Au-b{\|}_{2}^{2}+\;\alpha_{1}\|Du-p{\|}_{1}+\;\alpha_{2}\|Ep{\|}_{1}\\ \;+\;\alpha_{3}(\|u{\|}_{1}-\beta \langle u,q\rangle ). \end{array}$$

Next we apply the DCA (Tao and An, 1997) to obtain the following two subproblems

(15) $$\left\{ \begin{array}{l} \;q\leftarrow u/\|u{\|}_{2},\;\\ (u,p)\leftarrow \underset{u,p}{\text{argmin}}\frac{1}{2}\|Au-b{\|}_{2}^{2}+\;\alpha_{1}\|Du-p{\|}_{1}\\ \;+\alpha_{2}\|Ep{\|}_{1}+\;\alpha_{3}(\|u{\|}_{1}-\beta \langle u,q\rangle ). \end{array}\right.$$

In particular, the second subproblem can be solved efficiently using ADMM. By the change of variables, it can be further written as

$$\begin{array}{l} \underset{u,p,x,y,z}{\min}\frac{1}{2}\|Au-b{\|}_{2}^{2}+\;\alpha_{1}\|x{\|}_{1}+\;\alpha_{2}\|y{\|}_{1}+\;\alpha_{3}(\|z{\|}_{1}-\beta \langle z,q\rangle )\\ \text{ subject to }x=Du-p,\;y=Ep,\;z=u. \end{array}$$

By introducing the scaled multipliers $\tilde{x},\tilde{y},\tilde{z}$, we have the following augmented Lagrangian function

$$\begin{array}{ll} \begin{array}{l} L(u,p,x,y,z,\tilde{x},\tilde{y},\tilde{z})=\frac{1}{2}\|Au-b{\|}_{2}^{2}+\;\alpha_{1}\|x{\|}_{1}+\;\alpha_{2}\|y{\|}_{1}\\ \;+\;\alpha_{3}(\|z{\|}_{1}-\beta \langle z,q\rangle )\\ \;+\frac{\rho }{2}(\|Du-p-x{\|}_{2}^{2}+\;2\langle Du-p-x,\tilde{x}\rangle \;+\|Ep-y{\|}_{2}^{2}\\ \;+\;2\langle Ep-y,\tilde{y}\rangle \;+\|u-z{\|}_{2}^{2}+2\langle u-z,\tilde{z}\rangle ) \end{array} & . \end{array}$$

Note that this version is equivalent to the standard augmented Lagrangian function up to scaling of multipliers. We group the variables *u, p, x, y, z* into three blocks, i.e., *u*, *p* and (*x, y, z*). Then the ADMM yields the following algorithm

(16) $$\left\{ \begin{array}{l} u\leftarrow \underset{u}{\text{argmin}}\;L(u,p,x,y,z,\tilde{x},\tilde{y},\tilde{z})\\ p\leftarrow \underset{p}{\text{argmin}}\;L(u,p,x,y,z,\tilde{x},\tilde{y},\tilde{z})\\ \left(x,y,z)\leftarrow \underset{x,y,z}{\text{argmin}}\;L(u,p,x,y,z,\tilde{x},\tilde{y},\tilde{z}\right)\\ \tilde{x}\leftarrow \tilde{x}+Du-p-x\\ \tilde{y}\leftarrow \tilde{y}+Ep-y\\ \tilde{z}\leftarrow \tilde{z}+u-z+\frac{\alpha_{3}\beta }{\rho }q \end{array}\right.$$

Moreover, *u* and *p* can be solved explicitly as follows

$$\left\{ \begin{array}{l} u={\left(A^{T}A+\rho (D^{T}D+I)\right)}^{-1}(A^{T}b+\rho D^{T}(p+x\\ \;-\tilde{x})+\rho (z-\tilde{z}))\\ p={\left(E^{T}E+I\right)}^{-1}(E^{T}(y-\tilde{y})+(Du-x+\tilde{x})). \end{array}\right.$$

In addition, due to the separability of variables, the (*x, y, z*)-subproblem boils down to three independent subproblems with respect to *x*, *y* and *z*, respectively, each of which has a closed-form solution represented by proximal operators. For example, the *z*-subproblem can be solved by using the proximal operator of ℓ_1_-norm

(17) $$\begin{array}{l} \underset{z}{\text{argmin}}\left\{\alpha_{3}\|z{\|}_{1}+\frac{\rho }{2}\|u-z+\tilde{z}+\frac{\alpha_{3}\beta }{\rho }q{\|}^{2}\right\}\\ \;={\text{prox}}_{\alpha_{3}/\rho }(u+\tilde{z}+\frac{\alpha_{3}\beta }{\rho }q). \end{array}$$

where prox_γ_(*x*) = sign(*x*)⊙max{|*x*|−γ, 0} with componentwise multiplication ⊙, also known as shrinkage operator. Combining DCA for problem Equation (15) and ADMM for the (*u, p*)-subproblem, we obtain the algorithm summarized in Algorithm 1.

**Algorithm 1:** **Algorithm 1** s-SMOOTH EEG Reconstruction Algorithm

**Input:** the data *b*, the sensing matrix *A*, difference operators *D, E*, parameters α_1_, α_2_, α_3_ > 0 and β ∈ [0, 1], the maximal number of iterations for the outer loop *N*_*out*_, and the maximal number of iterations for the inner loop *N*_*in*_.
**Output:** the reconstructed *u*_*o*_.
**if** β = 0 **then**
*N*_*out*_ ← 0
**end if**
**Initialize** *u*_*o*_ = **0**.
**for** 1 to *N_out_* **do**
**if** *u_o_* = **0 then**
*q* ← **0**
**else**
*q* ← *u_o_*/||*u_o_*||_2_
**end if**
**Initialize** *p, x, y, z*, $\tilde{x}$, $\tilde{y}$, $\tilde{z}$ as zero vectors
**for** 1 to *N_in_* **do**
$\begin{array}{l} u\leftarrow {\left(A^{T}A+\rho (D^{T}D+I)\right)}^{-1}[A^{T}b+\rho D^{T}(p+x\\ \;-\;\tilde{x})+\rho (z-\tilde{z})] \end{array}$
$p\leftarrow {\left(E^{T}E+I\right)}^{-1}\left[E^{T}(y-\tilde{y})+(Du-x+\tilde{x})\right]$
*x* ← prox_α_1/ρ__(*Du – p +* $\tilde{x}$)
*y* ← prox_α_2/ρ__(*Ep* + $\tilde{y}$)
*z* ← prox_α_3/ρ__(*u* + $\tilde{z}$ + $\frac{\alpha_{3}\beta }{\rho }q$)
$\tilde{x}$ ← $\tilde{x}$ + *Du* – *p* – *x*
$\tilde{y}$ ← $\tilde{y}$ + *Ep* – *y*
$\tilde{z}$ ← $\tilde{z}$ + *u* – *z* + $\frac{\alpha_{3}\beta }{\rho }q$
**end for**
*u_o_* ← *u*
**end for**

Note that in this study the entire matrix *A* is scaled by multiplying 10^5^ in order to reduce round-off errors. Algorithm 1 terminates when either the maximal number of iterations or the minimal relative change is reached. Note that there are two loops in the algorithm: outer and inner loop. In our experiments, the maximum number of iterations for each inner loop is set to be 40, and the maximum number of outer loop is set to be 10. The algorithm will also be halted if the relative change of *u* is smaller than 10^−3^. Here the relative change of *u* is defined as

(18) $$u_{change}=\frac{\|u_{new}-u_{old}{\|}_{2}}{\|u_{old}{\|}_{2}}.$$

In general, ADMM is simple to implement with linear convergence even if part of the objective function is non-differentiable. Our empirical experience shows that the ℓ_1−2_ regularization further promotes faster convergence of the algorithm than its ℓ_1_-regularized counterpart.

#### 2.2.5. Parameter selection

In the proposed Algorithm 1, the regularization parameters α_1_, α_2_, α_3_ are selected to make a balance between smoothness and sparsity. Based on our large numbers of experiments, the optimal parameter selection does not change significantly as the source number, size or configuration changes. For different noise levels, the regularization parameters need to be tuned smaller when SNR increases. Table 1 lists all values of α_1_ that we use for the synthetic data sets with SNR between 0 and 30 dB. For simplicity, we set α_2_ to be equal to α_1_. A more detailed discussion about the influence of ratio α_2_/α_1_ on the reconstruction results can be found in (Papafitsoros and Valkonen, 2015). For α_3_, we find that the performance of the proposed method is not sensitive to α_3_ as long as it is in the range of α_3_ = 0.1 ~ 0.5α_1_. Figure 5 illustrates the source reconstruction results with different values of α_3_, where we can see that the results look very similar. By taking a careful look at the bottom source, one can see that α_3_ = 0.1α_1_ yields slightly under-focalized result, while α_3_ = 0.4α_1_ yields slightly over-focalized result, so α_3_ = 0.2 or 0.3 α_1_ provides results closest to the ground truth. In our experiment, we fix α_3_ to be 0.3α_1_ in all test cases. As for the parameter ρ, which controls the convergence speed of Algorithm 1, it is set to 10α_1_ by default. For real data sets, we use the same parameters for the same noise level as the synthetic data.

**Table 1:** **Parameter α_**1**_ used in different noise level**.

**SNR(dB)**	**0**	**5**	**10**	**15**	**20**	**25**	**30**
α_1_ (*10)	7	6	5	3	2	2	1

The parameter β in the ℓ_1−2_ regularization term varies from 0 to 1. When β = 0, the ℓ_1−2_ regularization becomes the ℓ_1_ regularization. In Figure 6, we study the effect of β on the source reconstruction results. Figure 6B shows the change of reconstruction error with different values of β, where we can see that the larger β is, the smaller the reconstruction error will be. When β = 1, the highest reconstruction accuracy is achieved. Figure 6C shows the change of the sparsity term as iteration increases. One can see that comparing to β = 0 (ℓ_1_ regularization), β = 1 (ℓ_1−2_ regularization) helps to promote sparsity. Notice that the sparsity term will decrease rapidly from one inner loop to another since the variable *q* is redefined in each outer loop. In our experiments, the maximal number of iterations at each inner loop is set to 40. At each inner loop, the solution becomes convergent and stable within the tolerance, so does the sparsity term. Then at the iteration 41, the updated *q* results in the refinement of the solution and a large drop of the sparsity term (Figure 6C). In sum, β = 1 not only helps reduce the reconstruction error, but also enhances the sparsity term. Therefore, we set β to 1 in the following study.

### 2.3. Experimental setup

#### 2.3.1. Synthetic data simulation

**Figure 4:**
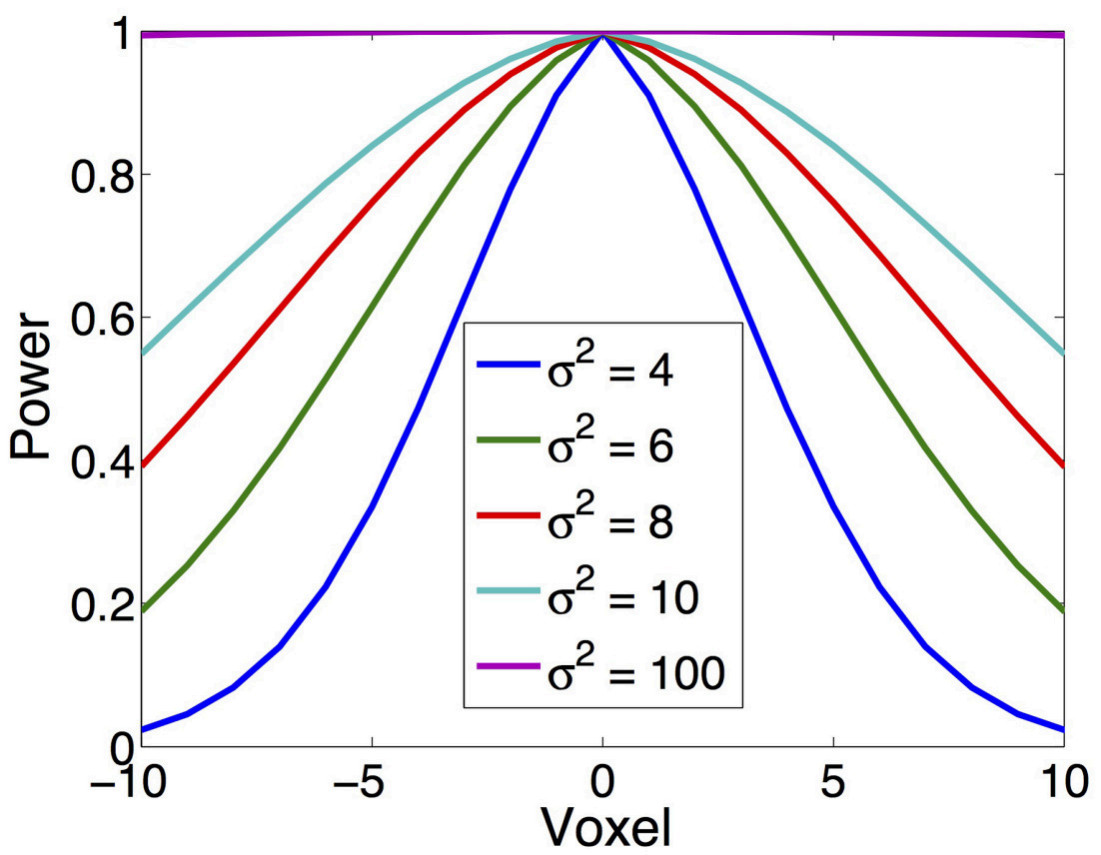
**Various source configurations (side view) with a shape of Gaussian function of different σ^**2**^**.

In our simulation, source is synthesized using the Gaussian-tapered patch. Firstly, a source center is seeded on the cortex surface, then its neighbors are gradually recruited to make a patch. Because the Gaussian function has bell shape, the source intensity distribution reaches a peak at the center and gradually decreases to zero as it moves away from the center. To model different source configurations, we use Gaussian functions with different variations (σ^2^), illustrated in Figure 4. As σ^2^ goes to infinity, the intensity of the source decays more and more slowly from the center to its neighbors and approximates the constant function.

In addition to various source configurations, we test a variety of sources with different sizes. Specifically, we use the sources containing 100~300 triangular voxels, which corresponds to 1.4~2.2 cm in radius. To study the sensitivity of the result to the measurement noise, we add i.i.d. additive white Gaussian noise to each channel. We also study the influence of the brain noise by adding i.i.d. Gaussian additive noise to the voxel space. As a widely used criterion for noise level measurement, the signal-to-noise ratio (SNR) is defined as

$$SNR=10\log_{10}\frac{P_{signal}}{P_{noise}},$$

where *P*_*signal*_ and *P*_*noise*_ are the power of the signal and the noise, respectively. In our simulation, SNR is set to 20 dB by default. The effect of different noise levels is also studied by using signals of SNR 0~20 dB. The synthetic signal is normalized to make sure that the amplitude of the signal falls into the range from 10 to 100 μ*V*, which is the typical EEG signal amplitude of an adult human (Aurlien et al., 2004). For synthetic data, we use the head model template provided by Fieldtrip (Oostenveld et al., 2003), where the number of voxels *M* is equal to 10240.

#### 2.3.2. Real data collection

To evaluate the performance of the proposed method in realistic scenario, we collected two P300 event-related potentials (ERPs) via auditory and visual oddball paradigms, in which a subject detected an occasional target stimulus in a regular train of sensory stimuli. The experiment was conducted with the approval of institutional review board at Hualien Tzu Chi General Hospital, Taiwan (IRB 101-102) with written informed consent from the subject.

P300 is a positive peak occurring about 300 ms or more after a stimulus (Linden, 2005), which reflects information processing associated with attention and memory. In the auditory stimulation setting, two audio signals of 1500 Hz (target, 40 trials) and 1000 Hz frequency (non-target, 160 trials) were randomly presented to the subject. In the visual stimulation setting, two different pictures of a fierce shark (40 trials) and of an old man (160 trials) were randomly presented to the subject. The subject was required to detect the targets by silently counting these events. A 64 channels EEG machine (ANT Neuro, Enschede Netherlands) was used to record the neural signals. The EEG data was sampled at 512 Hz, filtered by a band pass filter of 0.5–30 Hz and was referenced to the average of all channels. In the end, the average was taken across the trials in order to improve the SNR, and the difference between the target and non-target was used for source localization.

In addition to EEG data, high-resolution MRI data (General Electric, Waukesha, WI, USA) were obtained from the subject for realistic head model construction (Oostenveld et al., 2011). We first segmented the head into three layers, i.e., scalp, skull and brain, and then constructed a triangular mesh for each layer (Oostendorp and van Oosterom, 1991; Fuchs et al., 2002). The cortex surface was also triangulated into a fine mesh with 16384 triangles, each corresponding to a potential dipole source. Finally, BEM (Oostendorp and van Oosterom, 1991; Fuchs et al., 2002) was used to calculate the lead field matrix.

### 2.4. Quantitative metric

For synthetic data, in order to quantitatively evaluate the performance of an EEG source imaging method, we use the following three criteria to evaluate the results from different perspectives:

1. *Total reconstruction error* (TRE), which measures the relative difference between the true source and the reconstructed one (Im et al., 2003). The smaller the TRE is, the higher reconstruction accuracy the brain image will have. TRE is defined as
  $$TRE=\frac{\|\hat{u}-u{\|}_{2}}{\|u{\|}_{2}},$$
  where *u* is the true source, *û* is the reconstructed source. Note that TRE has no units since it is a relative value.
2. *Localization error* (LE), which measures the distance between the peaks of the true source and the reconstructed one (Im et al., 2003; Molins et al., 2008). Suppose that there are *k* underlying sources, and *LE*_*k*_ is the localization error of the *k*-th source, then LE is defined as the average localization error of all the sources. In order to define *LE*_*k*_, let *I*_*k*_ be a set of voxel indices that are spatially closest to the peak of the *k*-th source (the voxels with intensity less than 10% of the global maximum are not considered), and *d*_*ki*_ be the distance between the *i*-th voxel to the peak of the *k*-th true source. Then *LE*_*k*_ and LE can be expressed as
  $$LE=\frac{1}{K}\sum_{k}LE_{k},\;LE_{k}=\{d_{ki}|i=\underset{i^{\prime }\in I_{k}}{\text{argmax}}\|u_{i^{\prime }}{\|}_{2}\}.$$
3. *Degree of focalization* (DF), which describes how focal the reconstructed source is. It is defined as the energy ratio between the reconstructed and the true source in the true source area (Im et al., 2003)
  $$DF=\frac{\|{\hat{u}}_{S}{\|}_{2}^{2}}{\|u_{S}{\|}_{2}^{2}},$$
  where *u*_*S*_ is *u* restricted to the true source area *S*. The higher the DF is, the more focalized the reconstructed source will be. A perfect reconstruction has a DF of 100%.

### 2.5. Computational cost

In Algorithm 1, the two least squares subproblems involve matrix inverse which is computationally intensive. Instead of computing inverses of *P* = *A*^*T*^*A* + ρ(*D*^*T*^*D* + *I*) and *Q* = *E*^*T*^*E* + *I* directly, we apply the Cholesky decomposition and then solve linear systems using backward/forward substitution, i.e., mldivide in MATLAB. In addition, since the construction of *P* and *Q* does not depend on the time points, we can further reduce computational time by performing Cholesky decomposition once and saving results for all time points. For instance, when using 10240 voxels and running 100 iterations, the running time on a desktop with 3.4 GHz CPU and 16G memory using MATLAB 2014b is reduced from 3.5 min to 1.8 min.

Further, if we reduce the number of voxels to 6000, it takes about 11 s to run 100 iterations, and only 6.4 s if the matrices are pre-computed. If further decreasing the voxel number to be 2000, the computation time is reduced to 1.2 s, or 0.9 s with pre-computed matrices. Compared to relevant work (Haufe et al., 2008; Chang et al., 2010; Sohrabpour et al., 2016), the proposed algorithm has reduced the computational cost significantly.

## 3. Results

In this section, we evaluate the performance of the proposed method by conducting experiments on various synthetic data sets and two real data sets.

### 3.1. Synthetic data results

We compare the proposed method s-SMOOTH with four representative source localization methods in the literature: MNE, sLORETA, minimum ℓ_1_ method (“L1" for short) and TV-ℓ_1_. Figure 7 shows the reconstructed brain image of three synthetic sources, where the source intensity is scaled to be in [0 1]. A threshold is set at 20% of the maximum intensity, i.e., voxel intensity less than the threshold will be set to 0, so as to obtain a better visualization. For MNE and sLORETA which are minimum ℓ_2_ methods, one can see the reconstructed sources are spread out with a lot of spurious sources around the sources. The intensity of adjacent voxels has large jumps since these two methods do not consider the spatial relation between neighboring voxels. Regarding L1 method, the focalization of the reconstructed source is greatly improved. However, the sources are over-focused that only a few voxels are included in the area of the true sources. Compared to L1 method, the TV-ℓ_1_ method successfully recovers the extent of sources, but fails to reflect the intensity variation of the sources, as we can see that the intensity of the current density is almost uniform in each source region. In contrast, the proposed method not only eliminates the spurious sources, recovers the extent of the sources, but also provides a smooth result which reflects the magnitude variation of the current density.

**Figure 5:**
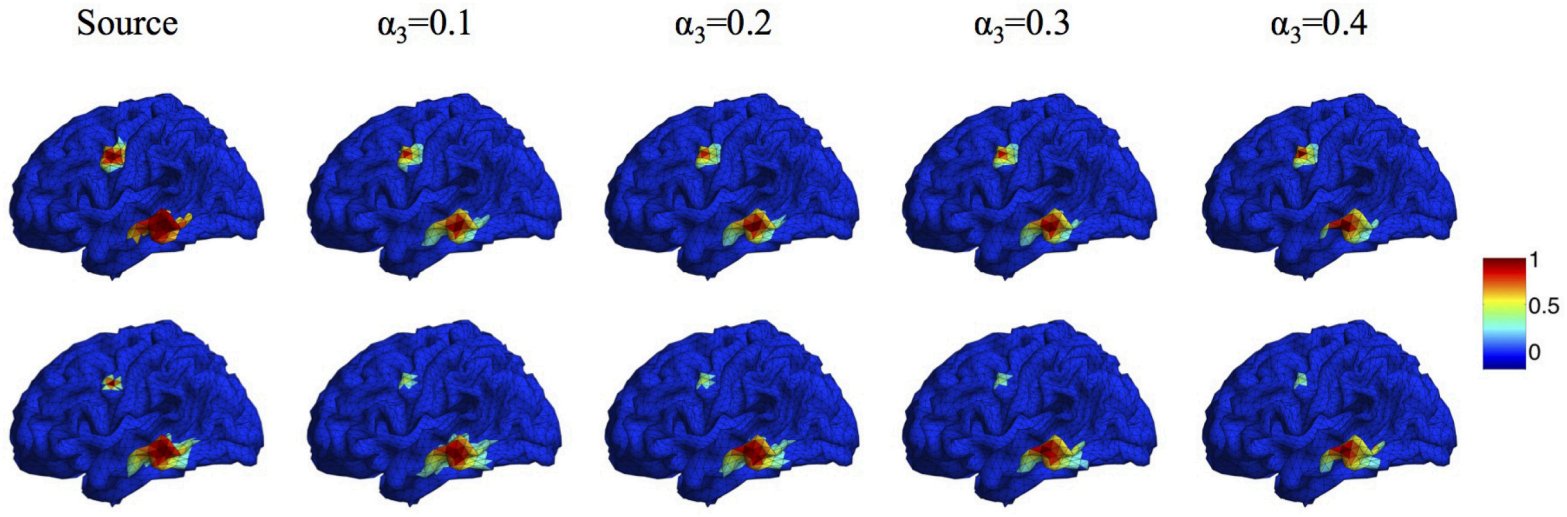
**Source localization results with different α_**3**_. Top:** two sources with different configurations. **Bottom:** two sources with different sizes.

**Figure 6:**
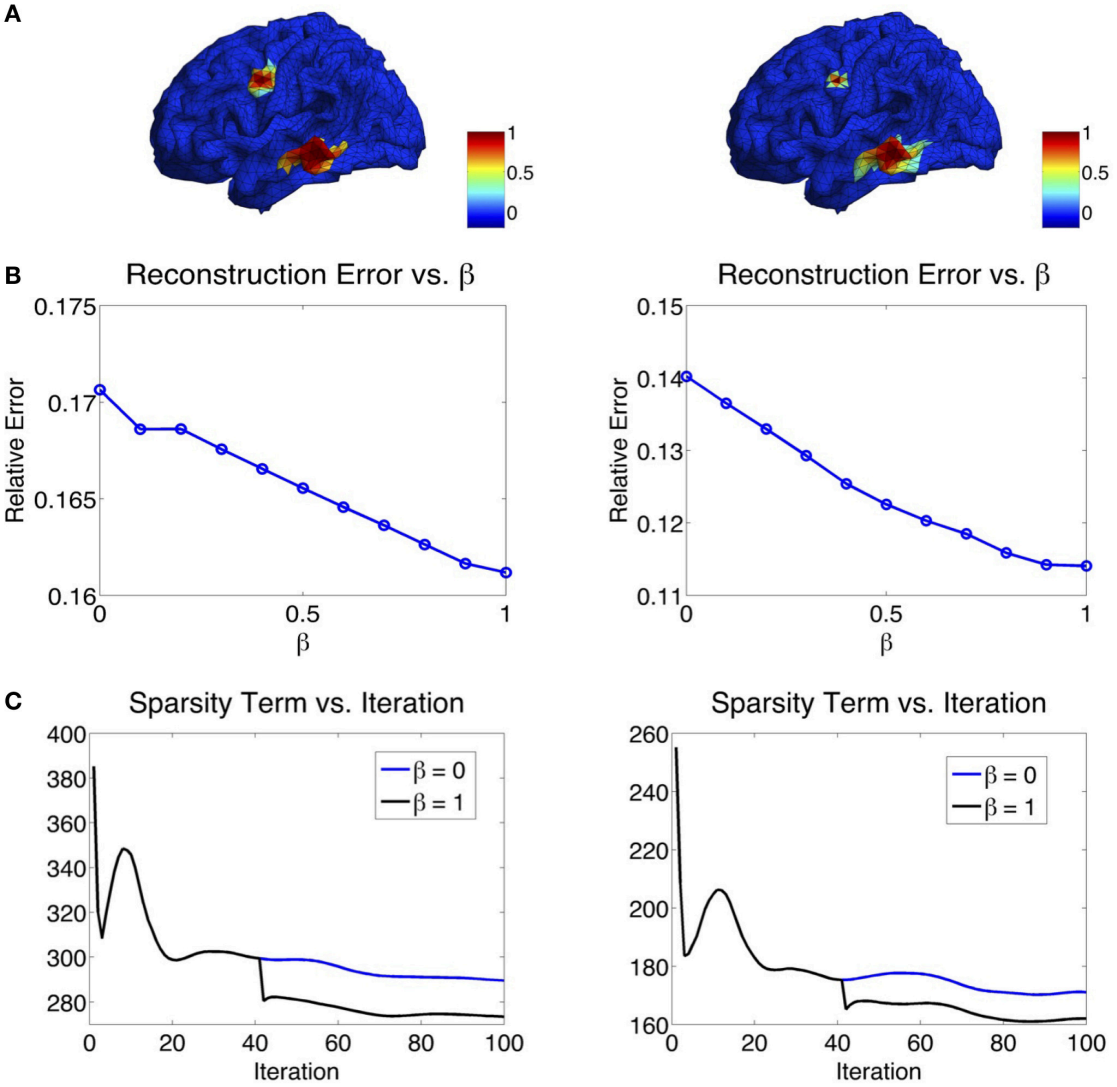
**(A)** Two simulated sources. **(B)** Influence of β on the reconstruction error. The larger the β, the smaller the reconstruction error will be. **(C)** Influence of β on the sparsity term. β = 1 enhances the sparsity compared to β = 0.

**Figure 7:**
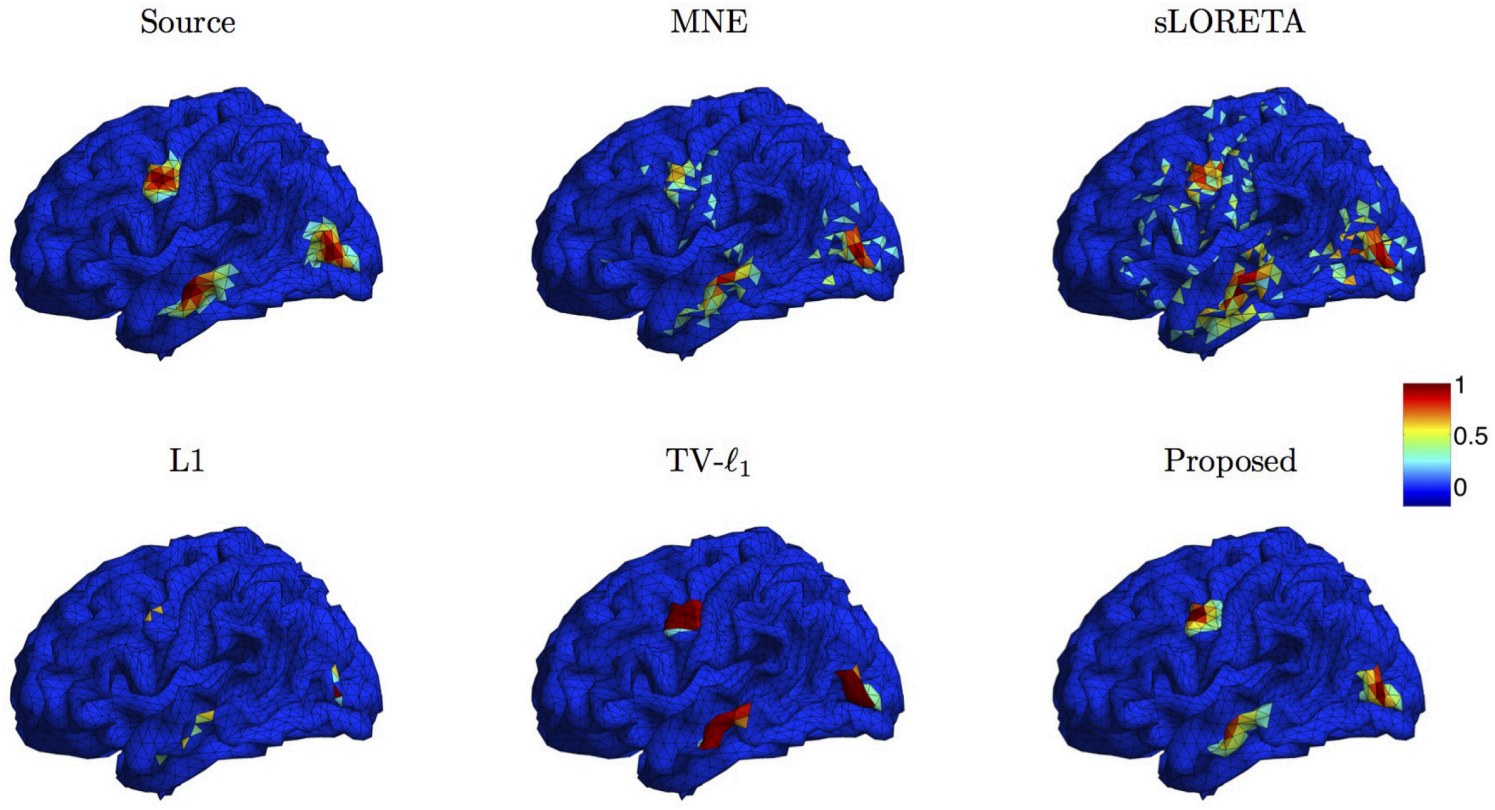
**Source localization results of various methods on synthetic data with three sources**.

### 3.2. Sensitivity study

In this section, we investigate the sensitivity of the proposed method to various factors both qualitatively and quantitatively.

#### 3.2.1. Influence of measurement noise level

**Figure 8:**
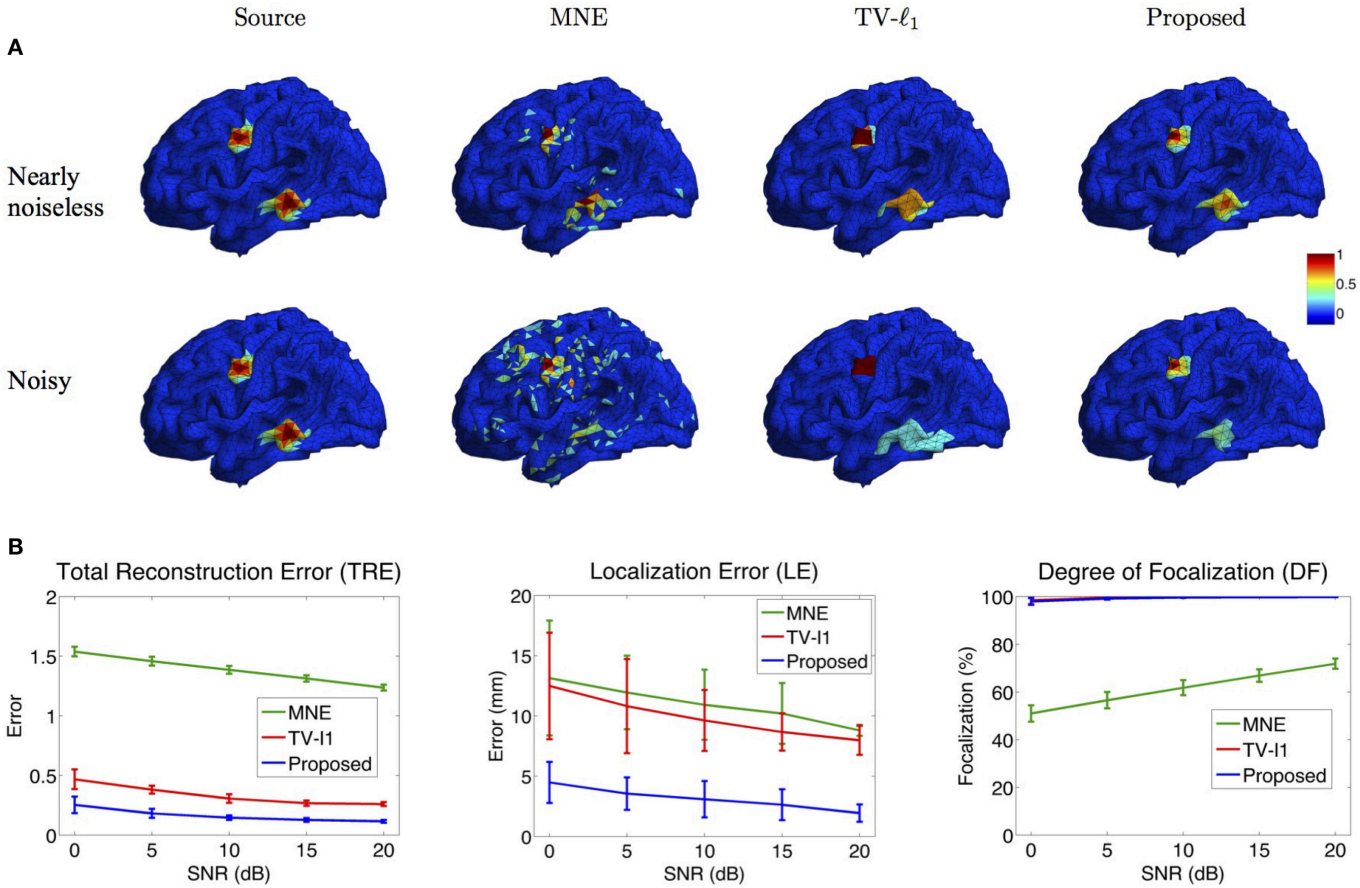
**Influence of measurement noise. (A)** Source localization results in the nearly noiseless (30 dB) and noisy (0 dB) cases. **(B)** Quantitative evaluation of various methods under different measurement noise levels. The plots show the average results across 50 repeats, where the error bar represents standard deviation.

Figure 8A illustrates the source localization results of two sources in nearly noiseless (30 dB) and noisy (0 dB) cases. In the nearly noiseless case, MNE successfully locates these two sources but produces a few spurious sources. For TV-ℓ_1_ method, although we can see a little magnitude variation in the edge of the sources, the main area of the sources still shows almost uniform current density distribution. Compared to the other two methods, the proposed method shows the closest result to the ground truth, where the magnitude of the current density varies smoothly from the peak to its neighbors. From the noisy case, one can see that the imaging result is sensitive to measurement noise, especially for the bottom source. MNE shows a lot more spurious sources than the nearly noiseless case even after thresholding. The TV-ℓ_1_ method shows an enlarged coverage of the bottom source compared to the ground truth. In addition, one can see that the source intensity becomes more flat in the noisy case. The proposed method is more robust to the noise with the coverage of the bottom source shrinks slightly.

To quantify the influence of noise levels on the source reconstruction performance, we test various noise levels and evaluate the results with the criteria defined in Section 2.4. In order to avoid inconsistency due to different noise configurations, we repeat the experiment 50 times by adding random noise and display the averaged result and the standard deviation in Figure 8B. Generally, the performance of all the methods is improved as SNR increases. From the TRE plot, one can see that our method has the smallest total reconstruction error compared to the other two methods. The LE plot shows that the proposed method has the smallest localization error. Compared to the proposed method, the TV-ℓ_1_ method has relatively large localization error since it tends to produce an almost uniform current density and thereby has difficulty locating the peak of the source. In the DF plot, both TV-ℓ_1_ method and the proposed method show very high focalization degree, this is because they incorporate ℓ_1_ or ℓ_1−2_ regularization to impose sparsity on the source current density. Taken together, the proposed method shows good performance for all three quantitative criteria at every noise level.

#### 3.2.2. Influence of brain noise level

**Figure 9:**
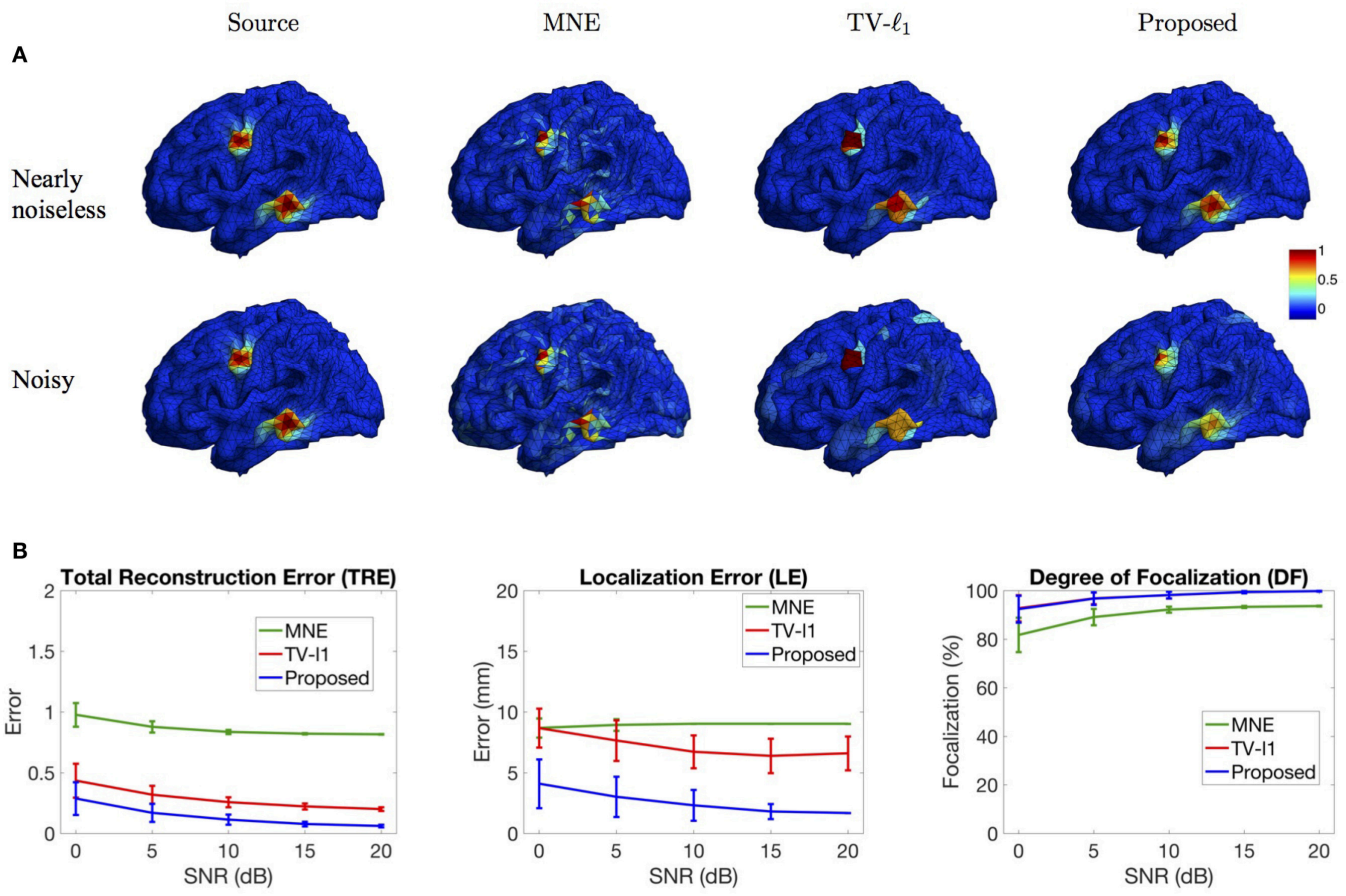
**Influence of brain noise. (A)** Source localization results in the nearly noiseless (30 dB) and noisy (0 dB) cases. **(B)** Quantitative evaluation of various methods under different brain noise levels. The plots show the average results across 50 repeats, where the error bar represents standard deviation.

In this section, we study the influence of brain noise by adding i.i.d. Gaussian additive noise to each voxel. Figure 9A shows the source imaging results in the nearly noiseless (30 dB) and noisy (0 dB) cases. Note that in this figure the imaging results are not thresholded so as to better visualize the influence of brain noise. In the nearly noiseless case, MNE produces much less spurious sources under the brain noise than under the measurement noise (Figure 8A), indicating that the spurious sources are mainly due to the measurement noise. For the TV-ℓ_1_ method, the reconstructed intensity distribution is generally piecewise constant, but we can see that the intensity variation is larger than the result in Figure 8A. The proposed method produces an accurate source intensity distribution that is very close to the ground truth. In the noisy case, generally the performance of all the methods is affected by the noise. The MNE result shows more background activities due to the high level of noise. The TV-ℓ_1_ result shows smaller intensity variation than the nearly noiseless case. For example, for the bottom source, we can see four different intensity colors in the nearly noiseless case, but only two different intensity colors in the noisy case. Compared to the TV-ℓ_1_ method, the proposed method provides a smoother result. We can see that the source intensity is weakened due to the high noise level.

Figure 9B further quantifies the results using different noise levels. The TRE plot shows that the proposed method has the smallest reconstruction error. In addition, by comparing to the result in Figure 8B with the same noise level, one can see that the reconstruction error under brain noise is smaller, which is consistent with the visualization result. The LE plot shows that the proposed method has the smallest localization error. It is worth noting that the localization errors of all the methods are smaller than those with measurement noise (Figure 8B). Finally, in the DF plot, both the proposed method and the TV-ℓ_1_ method achieve high focalization degree. The focalization degree for MNE is much higher than that under measurement noise. In summary, we observe that the brain imaging result is less sensitive to brain noise than to measurement noise. The proposed method demonstrates robust performance under various levels of brain noise.

#### 3.2.3. Influence of source size

**Figure 10:**
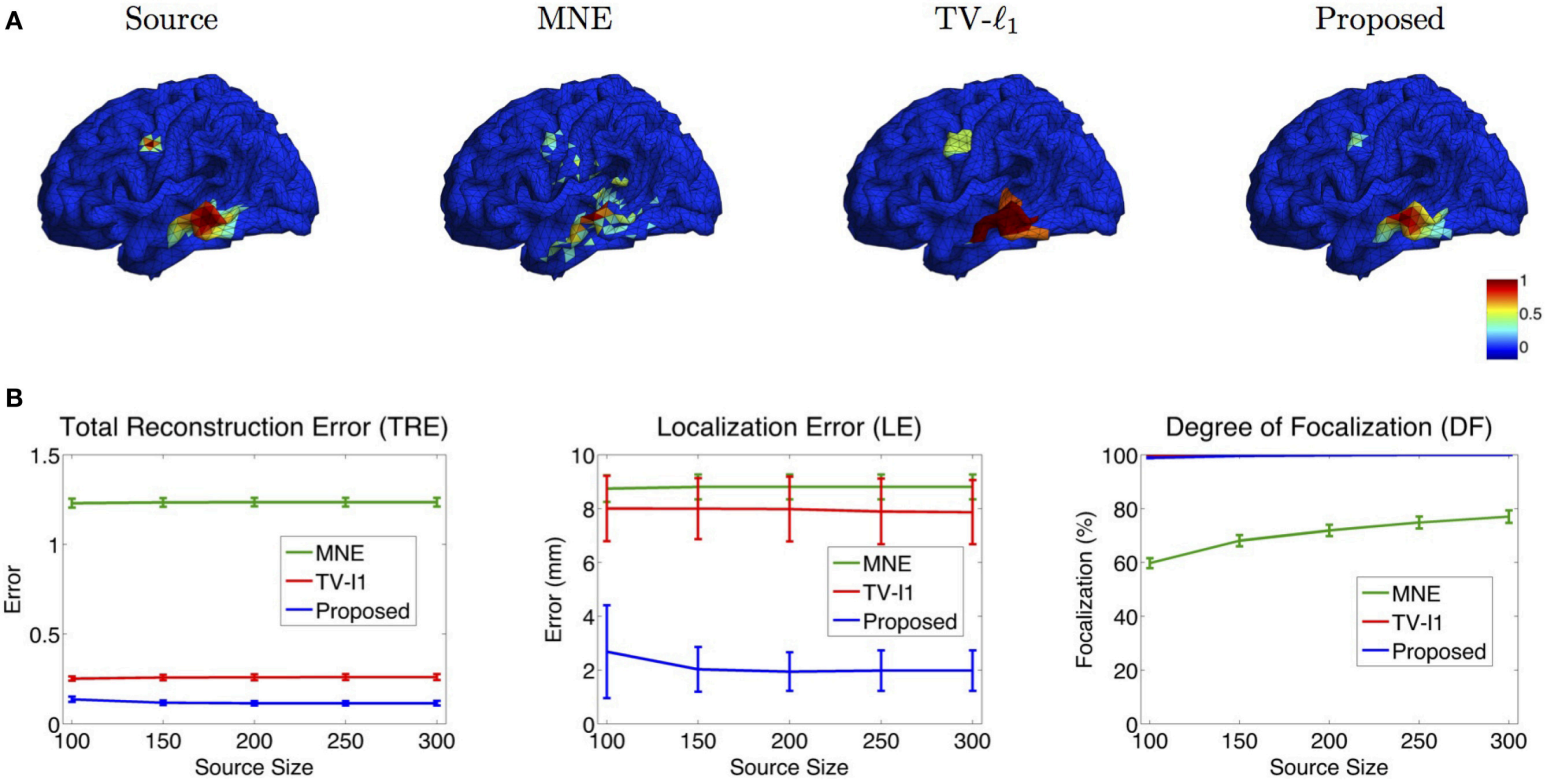
**(A)** Source localization results of various methods for two sources with different source sizes. **(B)** Quantitative evaluation of various methods with different source sizes. The average result of 50 repeats is shown in the plots, where the error bar represents the standard deviation.

In addition to noise level, we also investigate the influence of the source size on the reconstruction results. Figure 10A illustrates the reconstructed brain image with two sources of different sizes. In MNE, although it locates these two sources at the approximate locations, however, it is difficult to differentiate the smaller source from the large numbers of spurious sources. TV-ℓ_1_ method recovers both sources clearly without spurious sources, but the coverage of the reconstructed sources is enlarged, especially for the small source on the top. Additionally, it fails to recover the intensity variation of the source in space. In contrast, the proposed method accurately reconstructs the size and intensity variation of these two sources.

Figure 10B shows the quantitative results of various source sizes, where the *x*-axis represents the number of voxels contained in the simulated sources. TRE plot shows that the proposed method has the smallest reconstruction error, which is insensitive to the source size. In the LE plot, the proposed method shows the smallest localization error. As the source size increases, its localization error becomes slightly smaller, which implies that the proposed method has advantages of dealing with larger sources. The TV-ℓ_1_, by contrast, shows relatively large localization error due to the uniform intensity of the reconstructed source. In the DF plot, the proposed method demonstrates very high focalization degree. In summary, the proposed method shows consistent outstanding performance over the other two methods regardless of the source size.

#### 3.2.4. Influence of source configuration

**Figure 11:**
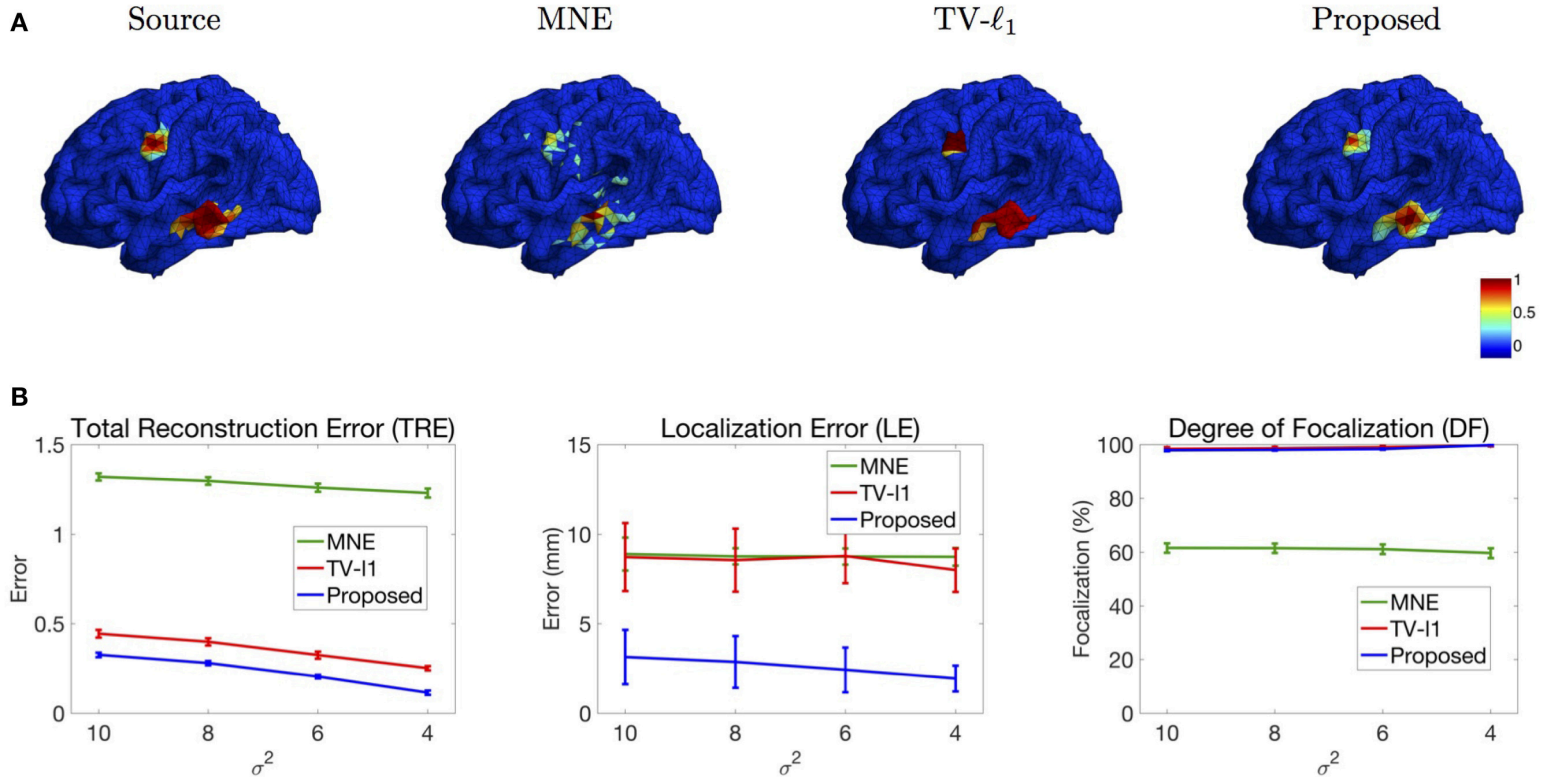
**(A)** Source localization results of two sources data with different configurations. **(B)** Quantitative results of various methods with different source configurations (σ^2^). The average result of 50 repeats is shown in the plots, where the error bar represents the standard deviation.

We study the performance of the proposed method using sources with different decay speeds (see Figure 4). In Figure 11A we show two sources of different configurations: the top source decays fast as it goes far from the center while the bottom source decays slowly. From the reconstruction results, one can see that the MNE is not able to tell the configuration difference between these two sources. The TV-ℓ_1_ method models the source intensity to be piecewise constant, so both of the reconstructed sources decay very slowly. As for the proposed method, we can tell that the bottom source decays more slowly than the top one.

We further evaluate the performance of the methods with different source configurations quantitatively. In Figure 11B, the *x*-axis represents the variance σ^2^ of the Gaussian function (Figure 4), so the source intensity decays faster and faster from left to right. The TRE plot shows that the proposed method has the smallest reconstruction error among all the methods. By comparing the results of different variance σ^2^, one can see that the proposed method favors smoother sources whose intensity decays faster, i.e., smaller σ^2^. In the LE curve, the proposed method shows much smaller localization error than the other two methods. Again, one can see that the smoother sources have smaller localization errors. Finally, the DF plot shows that the focalization degree does not rely on the source configurations too much. In sum, the proposed method outperforms the other two methods consistently for all three criteria. Compared to constant sources, it favors smoother sources.

#### 3.2.5. Influence of source location

**Figure 12:**
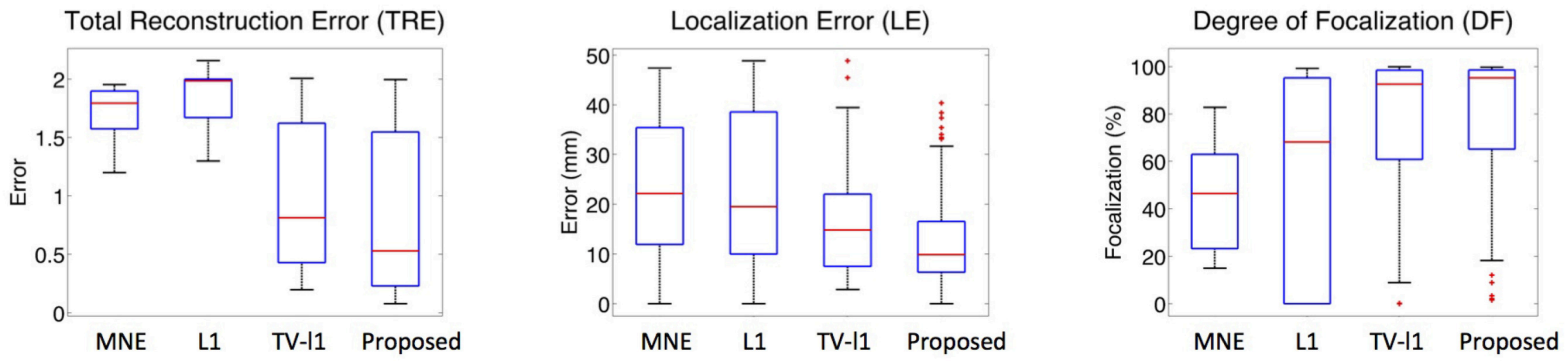
**Whisker plots of various methods at different source locations**. The red bar represents the median value of 50 random locations.

To systematically evaluate the performance of the proposed method for different source locations, we randomly select 50 locations in the whole source space, and test its average performance. Figure 12 displays the whisker plot of the quantitative results, where the lower quartile, median and upper quartile are shown. In TRE plot, the proposed method shows the best median reconstruction accuracy. The range of the results is relatively large which indicates the performance varies at different locations. The LE plot shows that the localization error of the proposed method has a median value of around 1 cm, which is the smallest among all the methods. In addition, the range of its localization error is also the smallest. From the DF plot, one can see that the median focalization degree of the proposed method is ~ 97% which is the highest. All in all, the proposed method shows the best average performance for different source locations among all the compared methods.

### 3.3. Real data results

We have also applied the proposed method to localize the generators of P300 ERPs. Although the neural generators of P300 remain imprecisely located, a consistent pattern of P300 sources has been shown by various techniques, such as intracranial recordings, lesion studies and fMRI-EEG combination, that the target-related responses locate in the parietal cortex and the cingulate, with stimulus specific sources in the superior temporal cortex for the auditory stimulation and in the inferior temporal, and superior parietal cortex for the visual stimulation (Linden, 2005). It is shown that there is a significant amplitude difference between target and non-target at latency of 300–400 ms for auditory stimulation and of 400–500 ms for visual stimulation (Linden et al., 1999).

**Figure 13:**
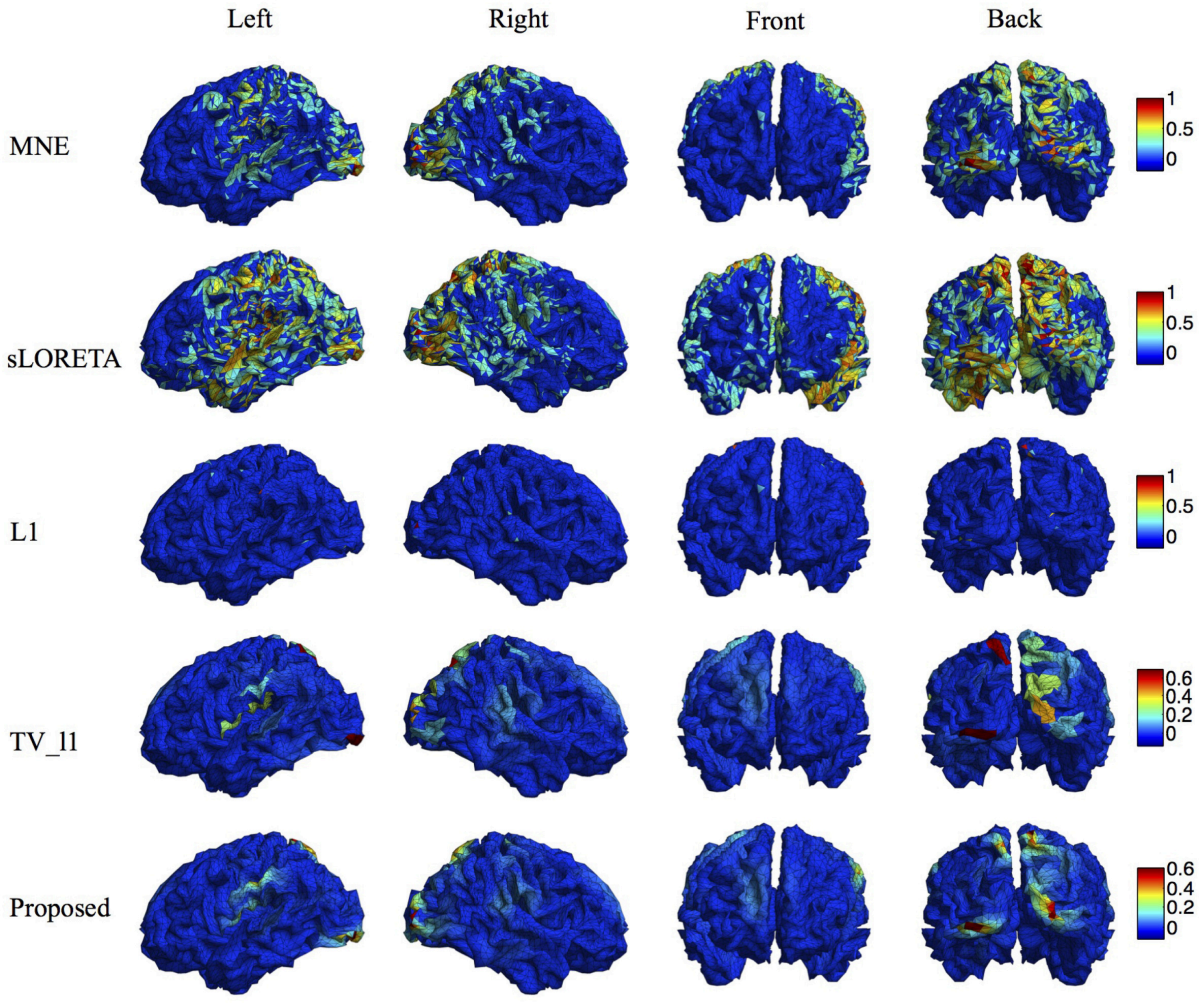
**Localization results of auditory P300 sources with different methods**.

**Figure 14:**
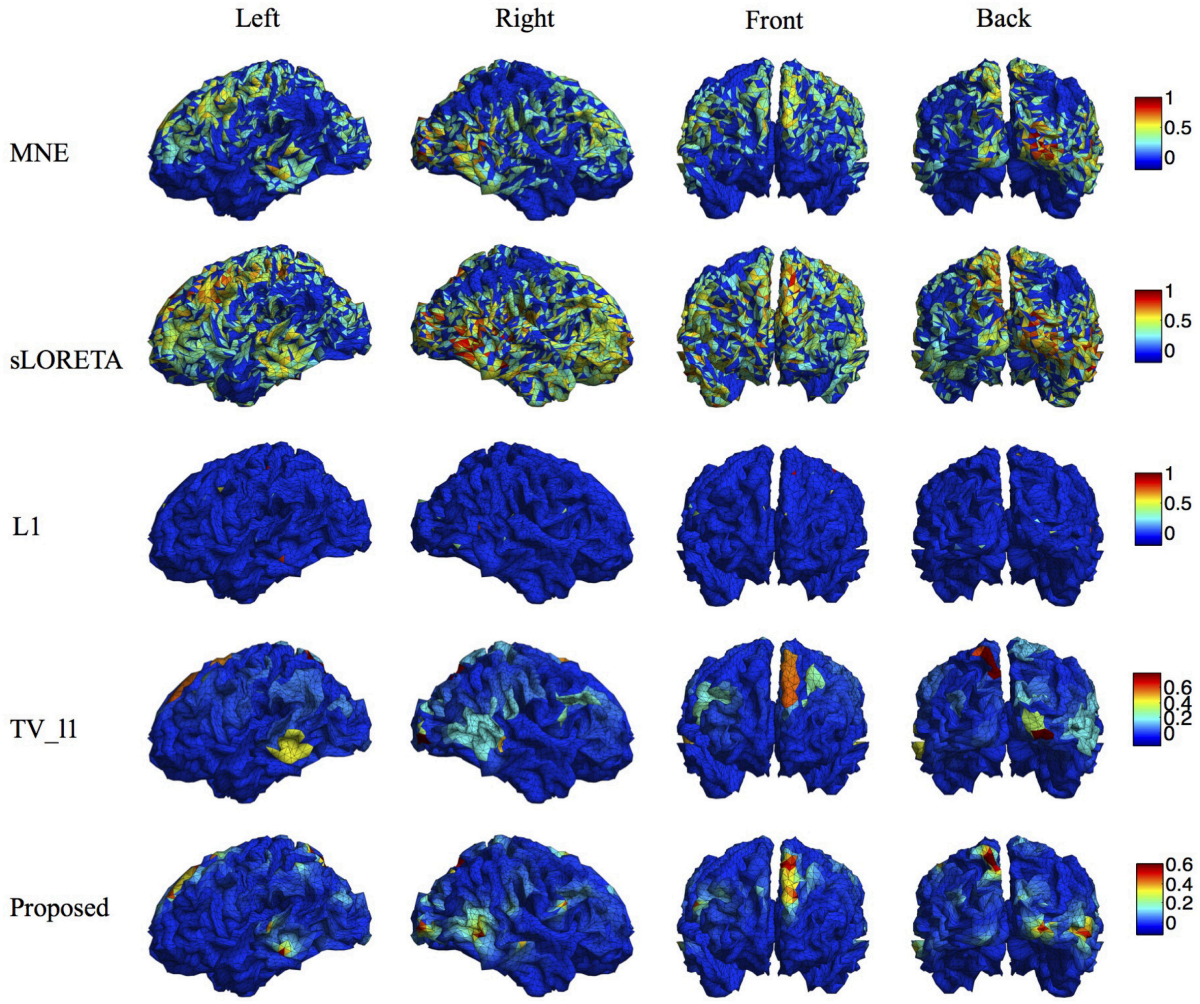
**Localization results of visual P300 sources with different methods**.

We compare the proposed method with various representative methods, including MNE, sLORETA, minimum ℓ_1_ method (“L1” for short), and TV-ℓ_1_. Among them, sLORETA has been widely used to localize the sources of P300 (Sumiyoshi et al., 2009; Bae et al., 2011; Machado et al., 2014) due to its high localization accuracy, which can be used as a rough reference. Figure 13 illustrates the P300 source localization results of auditory stimulation at the peak (312 ms). Since the results of MNE and sLORETA show low spatial resolution, a threshold is set at 20% of the maximum intensity to improve the visualization. One can see that the source localization results of different methods generally agree with each other. The sources from insula, superior temporal, temporo-parietal junction and parietal cortex are detected, which agree with previous literature (Linden et al., 1999; Mulert et al., 2004; Linden, 2005). The results of MNE and sLORETA are spread out with many spurious sources, and the extent of the sources is difficult to be identified. L1 method generates an over-focused result that only a few voxels are active in each source area. TV-ℓ_1_ produces a result with clearer extent, however the current density is piecewise constant in each source subregion. In contrast, our method provides a smooth result that reflects the intensity variation of the sources in space. Figure 14 shows the source localization results of visual stimulation at 438 ms, in which the sources in posterior temporal, parietal and mesial frontal cortices are found, which generally agrees with previous literature (Linden et al., 1999; Linden, 2005). One can see that the image resolution for MNE and sLORETA is very low, especially for sLORETA. L1 method only pinpoints a few active voxels and TV-ℓ_1_ provides an almost uniform current density in each source region. Compared to other methods, the proposed method demonstrates the capability of producing brain images with better smoothness and higher spatial resolution.

## 4. Discussion

In this study, we develop a novel EEG source imaging method aiming to accurately reconstruct the location, extent and magnitude variation of the current density distribution. The contributions of this work are threefold: (1) a vTGV regularization is defined, which incorporates the information of higher-order derivatives, therefore is able to enhance smoothness of the reconstructed brain image as well as reduce the staircasing artifacts; (2) a new ℓ_1−2_ regularization is introduced to the EEG source imaging field for the first time, which is able to reconstruct a sparser source than the widely used ℓ_1_ regularization; (3) an efficient algorithm is derived to solve the proposed model based on DCA and ADMM. The reconstructed brain image by the proposed method shows not only high location accuracy, but also high focalization degree.

Due to the ill-posedness of EEG inverse problem, the source image reconstruction relies on the modeling of the characteristics of underlying sources. MNE and sLORETA do not model the spatial relation between adjacent dipoles, thus the reconstructed current density distribution is not smooth and many spurious sources are generated. Minimum ℓ_1_-norm methods, such as MCE, assume the source to be highly focalized thus is not suitable for spatially extended sources. TV based methods assume the intensity of the source to be uniformly distributed in space, hence fail to reflect the intensity variation of the sources. This effect becomes more obvious when the regularization parameter increases, resulting in even more flat intensity distribution (Gramfort, 2009). By contrast, the proposed method s-SMOOTH assumes the intensity of the adjacent dipoles to be piecewise polynomial, resulting in a brain image which is very smooth that recovers the magnitude variation within a source precisely (Figure 7). The performance of the proposed method is evaluated under various noise levels, source sizes, source configurations and locations. The simulation results show that the source reconstruction result of s-SMOOTH is robust under different conditions. Quantitative results show that the performance of s-SMOOTH improves as the noise level decreases (Figures 8B, 9B), source size increases (Figure 10B) and current density distribution gets far from a constant function (Figure 11B).

**Figure 15:**
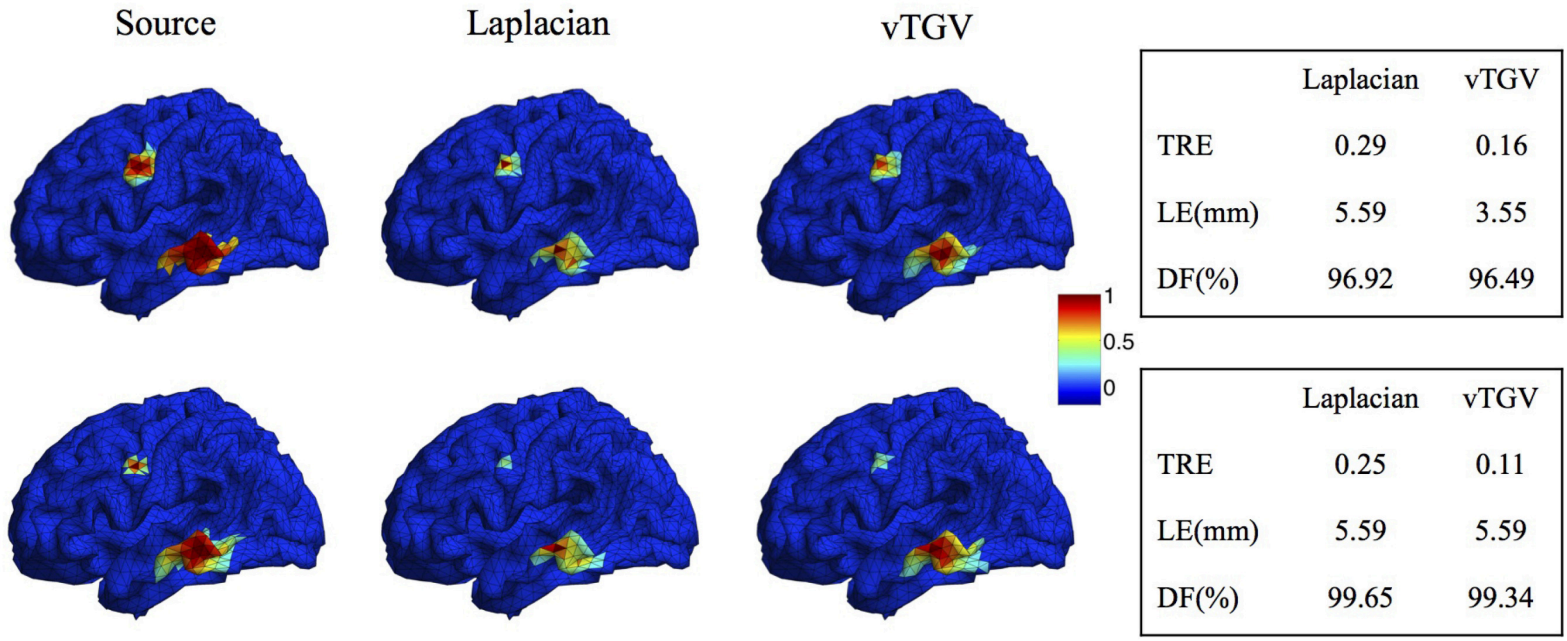
**Comparison of Laplacian and vTGV operator. Top:** two sources with different configurations. **Bottom:** two sources with different sizes. The left panel visualizes the source localization results. The vTGV operator provides accurate results with intensity distribution closer to the ground truth. The right panel shows the quantitative results.

The classical TGV assumes that the underlying image is piecewise polynomial (including piecewise constant, linear, quadratic, etc.) and thus imposes sparsity in high-order spatial derivatives. In this work we extend the TGV framework from Euclidean spaces to irregular surfaces and propose a novel second-order vTGV operator. A large number of simulation experiments with Gaussian-shaped sources show that it provides better results than the state-of-the-art methods. It is sufficient to use second order considering the computational cost and performance improvement. Note that the second-order TGV is mathematically different from the Laplacian operator (Bredies et al., 2010) used in previous methods, such as LORETA, FVR and CENT^*L*^. Laplacian operator has been widely used in EEG brain imaging (Pascual-Marqui et al., 1994; Haufe et al., 2008; Chang et al., 2010) due to its simple form. However, it only considers the unmixed second partial derivatives and does not involve the mixed partial derivatives. It assigns high weight to the central dipole and low weights to its neighbors, resulting in a very high peak in the center of the reconstructed source. In contrast, the proposed vTGV operator takes both unmixed and mixed partial derivatives into account and is able to recover fine details of brain images. Figure 15 compares the reconstructed brain image using a Laplacian operator and the proposed vTGV operator. One can see that the vTGV operator reconstructs the intensity variation of the sources more precisely. With the Laplacian operator, the reconstructed sources show a high peak in the center and the intensity decays very fast from the center (“over-smoothing” effect). Note that in this paper we treat each triangle as voxel, so each voxel has three neighbors. Accordingly, the weighting assigned to the central voxel by Laplacian operator is 1 and is -1/3 for its neighbors. In the case that each vertex is treated as voxel, this over-smoothing effect will become even more severe, since each vertex usually has 6 neighbors thus the weighing assigned to the neighbors will be only -1/6. From the quantitative results in the right panel of Figure 15, one can see that the vTGV operator is advantageous in both total reconstruction accuracy and localization accuracy. The focalization degree results are very close for both operators.

The ℓ_1_-norm regularization has been used in EEG source imaging to improve the focalization of the source for a long time (Matsuura and Okabe, 1995; Uutela et al., 1999; Huang et al., 2006; Ding and He, 2008). In this paper, we use the ℓ_1−2_ regularization instead of the ℓ_1_-norm regularization to enhance sparsity of the image. The ℓ_1−2_ regularization is a very recently proposed regularization technique which refines the ℓ_1_ regularization by taking the difference between the ℓ_1_ and ℓ_2_ norms. In this paper, we set the parameter β ∈ [0, 1]. When β is equal to 0, ℓ_1−2_ regularization becomes the ℓ_1_-norm regularization. We show that the reconstruction accuracy is improved as β increases, and it achieves the highest accuracy when β = 1 (Figure 6B). Therefore, we set the β to 1 in our experiments. Figure 6C shows that with β = 1, the sparsity of the image improves faster than β = 0, implying that the sparsity of the image is further enhanced using the ℓ_1−2_ regularization compared to ℓ_1_ regularization. On the other hand, if sparsity is fixed, ℓ_1−2_ regularization helps to accelerate the convergence of the optimization algorithm.

It is worth noting that the proposed objective function is a very general frame, which includes some related methods, e.g., L1, TV and TV-ℓ_1_, as its special cases by choosing proper parameters. For example, by setting the α_2_, *p* and β to be 0, it becomes the TV-ℓ_1_ method. By further setting the α_3_ to be 0, it becomes the TV method. On the other hand, if choosing α_1_, α_2_ and β to be 0, it becomes the L1 method. In addition, some relevant methods that combine two regularization terms (Haufe et al., 2008; Chang et al., 2010; Sohrabpour et al., 2016) usually describe the data fidelity by using an inequality constraint. Instead, we integrate this term into our objective function. This enables us to apply efficient optimization methods such as ADMM to derive a fast and robust algorithm. Compared to the optimization algorithms used in these methods (Haufe et al., 2008; Chang et al., 2010; Sohrabpour et al., 2016), the proposed algorithm in this paper is more efficient and robust, and it is also able to tackle large-scale problems. Further, with this type of problem formation, it is possible to adopt some computing techniques (Peng et al., 2015) to further accelerate the algorithm, which will be the future work.

For parameter selection, we provide some typical parameter values that work well in our experiment. Table 1 lists some values for α_1_ used in our experiments. For α_2_, we simply set it to be equal to α_1_. Note that it might provide a better result if α_2_ is further tuned. For α_3_, the parameter associated with the sparsity term, we show that the source reconstruction results are not sensitive to the choice of α_3_ as long as it is within the range 0.1 ~ 0.5α_1_. Specifically, we suggest to use α_3_ = 0.3α_1_. Notice that in this study we focus on spatially extended sources rather than point sources, therefore we assign relatively small weighting to the sparsity term. In the case that the underlying source is point source, larger weights can be assigned to the sparsity term (e.g., α_3_ = 100α_1_) so as to make the reconstructed source highly focalized. So far these parameters are tuned manually. In the future, the parameters could be selected in an automatic fashion by treating the parameters as unknown variables in the proposed model Equation (13) and then solving the corresponding optimization problem using the bilevel approach (Kunisch and Pock, 2013; Calatroni et al., 2015; Reyes et al., 2016).

In the present study, the EEG source imaging method works for each time point independently. In the future, the relationship between two contiguous time points could also be modeled so that the brain imaging is done in a spatiotemporal manner. Considering that the current source distributions between consecutive time points usually changes smoothly (Baillet and Garnero, 1997; Galka et al., 2004; Zhang et al., 2005), the temporal smoothness of the signal could be integrated into the proposed objective function to further improve the reconstruction accuracy (Ou et al., 2009; Gramfort et al., 2012).

## 5. Conclusion

In this paper, we propose a novel EEG inverse method *Sparsity and SMOOthness enhanced brain TomograpHy* (s-SMOOTH), which combines the vTGV and the ℓ_1−2_ regularizations to improve reconstruction accuracy for EEG source imaging. Considering the complicated geometries of the cortex surface, we define a vTGV regularization on a triangular mesh expressed as an infimal convolution form. The vTGV regularization enhances the high-order smoothness and thus is able to improve localization accuracy, while the ℓ_1−2_ regularization enhances the sparsity of the brain images. A series of simulation experiments with Gaussian-shaped sources show that the proposed s-SMOOTH is able to accurately estimate the location, extent and magnitude variation of the current density distribution. It also consistently provides better performance than other competitive methods in terms of quantitative criteria such as total reconstruction accuracy, localization accuracy, and degree of focalization. The test on two P300 data sets further shows the advantage of s-SMOOTH over state-of-art-methods in terms of brain image quality. Although this paper focuses on discussing EEG source imaging, the proposed method is equivalently applicable to MEG source imaging.

## Author contributions

YL and JQ have contributed to the conception and design of the work. YL has contributed to the analysis and interpretation of data, and the drafting of the work. YH has contributed to the acquisition of data. JQ, SO and WL have contributed to the interpretation of data. WL and YL have identified the need of a high-density brain imaging system using EEG modality, as well as initiated and defined the study approach accordingly. All authors have revised the work critically for important intellectual content, approved the version to be published, and agreed to be accountable for all aspects of the work in ensuring that questions related to the accuracy or integrity of any part of the work are appropriately investigated and resolved.

### Conflict of interest statement

The authors declare that the research was conducted in the absence of any commercial or financial relationships that could be construed as a potential conflict of interest. The proposed method was filed as part of a patent “UC-2016-151-2-LA-FP PCT/US2016/050452 Ultra-Dense Electrode-Based Brain Imaging System.”
